# Pre-exposure prophylaxis for preventing acquisition of HIV: A cross-sectional study of patients, prescribers, uptake, and spending in the United States, 2015–2016

**DOI:** 10.1371/journal.pmed.1003072

**Published:** 2020-04-10

**Authors:** Stephanie S. Chan, Andre R. Chappel, Karen E. Joynt Maddox, Karen W. Hoover, Ya-lin A. Huang, Weiming Zhu, Stacy M. Cohen, Pamela W. Klein, Nancy De Lew

**Affiliations:** 1 US Department of Health and Human Services (HHS), Office of the Assistant Secretary for Planning and Evaluation, Office of Health Policy, Washington, DC, United States of America; 2 Washington University School of Medicine in St. Louis, St. Louis, Missouri, United States of America; 3 HHS, Centers for Disease Control and Prevention, Division of HV/AIDS Prevention, Epidemiology Branch, Atlanta, Georgia, United States of America; 4 HHS, Health Resources and Services Administration, HIV/AIDS Bureau, Rockville, Maryland, United States of America; University of Washington Department of Global Health, UNITED STATES

## Abstract

**Background:**

In 2015, there were approximately 40,000 new HIV diagnoses in the United States. Pre-exposure prophylaxis (PrEP) is an effective strategy that reduces the risk of HIV acquisition; however, uptake among those who can benefit from it has lagged. In this study, we 1) compared the characteristics of patients who were prescribed PrEP with individuals newly diagnosed with HIV infection, 2) identified the specialties of practitioners prescribing PrEP, 3) identified metropolitan statistical areas (MSAs) within the US where there is relatively low uptake of PrEP, and 4) reported median amounts paid by patients and third-party payors for PrEP.

**Methods and findings:**

We analyzed prescription drug claims for individuals prescribed PrEP in the Integrated Dataverse (IDV) from Symphony Health for the period of September 2015 to August 2016 to describe PrEP patients, prescribers, relative uptake, and payment methods in the US. Data were available for 75,839 individuals prescribed PrEP, and findings were extrapolated to approximately 101,000 individuals, which is less than 10% of the 1.1 million adults for whom PrEP was indicated. Compared to individuals with newly diagnosed HIV infection, PrEP patients were more likely to be non-Hispanic white (45% versus 26.2%), older (25% versus 19% at ages 35–44), male (94% versus 81%), and not reside in the South (30% versus 52% reside in the South).Using a ratio of the number of PrEP patients within an MSA to the number of newly diagnosed individuals with HIV infection, we found MSAs with relatively low uptake of PrEP were concentrated in the South. Of the approximately 24,000 providers who prescribed PrEP, two-thirds reported primary care as their specialty. Compared to the types of payment methods that people living with diagnosed HIV (PLWH) used to pay for their antiretroviral treatment in 2015 to 2016 reported in the Centers for Disease Control and Prevention (CDC) HIV Surveillance Special Report, PrEP patients were more likely to have used commercial health insurance (80% versus 35%) and less likely to have used public healthcare coverage or a publicly sponsored assistance program to pay for PrEP (12% versus 45% for Medicaid). Third-party payors covered 95% of the costs of PrEP. Overall, we estimated the median annual per patient out-of-pocket spending on PrEP was approximately US$72. Limitations of this study include missing information on prescription claims of patients not included in the database, and for those included, some patients were missing information on patient diagnosis, race/ethnicity, educational attainment, and income (34%–36%).

**Conclusions:**

Our findings indicate that in 2015–2016, many individuals in the US who could benefit from being on PrEP were not receiving this HIV prevention medication, and those prescribed PrEP had a significantly different distribution of characteristics from the broader population that is at risk for acquiring HIV. PrEP patients were more likely to pay for PrEP using commercial or private insurance, whereas PLWH were more likely to pay for their antiretroviral treatment using publicly sponsored programs. Addressing the affordability of PrEP and otherwise promoting its use among those with indications for PrEP represents an important opportunity to help end the HIV epidemic.

## Abstract 2

Stephanie Chan and colleagues report on provision of medication for pre-exposure prophylaxis against HIV infection in the United States.

## Author summary

**Why was this study done?:**

- Pre-exposure prophylaxis (PrEP) is an effective biomedical prevention strategy that reduces the risk of HIV acquisition.
- However, uptake of this intervention among those who can benefit from it most has lagged.
- Prior studies lack information about PrEP patients’ race/ethnicity, geographic distributions at the metropolitan statistical area (MSA) level, sex, educational attainment, income, and patient and insurance payments in the United States.

**What did the researchers do and find?:**

- We analyzed a large, nationally representative prescription claims database from Symphony Health for the period of September 2015 to August 2016 in the United States.
- Compared to individuals with newly diagnosed HIV infection, PrEP patients were more likely to be male, non-Hispanic White, and less likely to live in the South.
- MSAs with relatively low uptake of PrEP were concentrated in the South.
- Compared to the types of payment methods that people living with diagnosed HIV (PLWH) used to pay for their antiretroviral treatment, PrEP patients were more likely to have used commercial health insurance and less likely to have used public healthcare coverage or a publicly sponsored assistance program.

**What do these findings mean?:**

- Many individuals who may benefit from being on PrEP are not currently receiving this HIV prevention medication, and those prescribed PrEP have a significantly different distribution of characteristics from the broader population that is at risk for acquiring HIV.
- Third-party payors covered much of the costs. For many insured patients, insurance coverage helped substantially offset the cost of PrEP medication.

## Introduction

HIV remains a significant public health concern in the United States. Currently, an estimated 1.1 million individuals are living with HIV [1]. Although new HIV diagnoses declined 4.3% from 41,942 in 2012 to 40,534 in 2016, progress has been uneven among different populations and geographic locations in the US and 6 dependent areas. Of new diagnoses in 2016, nearly 70% occurred among men who have sex with men and were disproportionately concentrated among minorities. The South accounted for both the highest proportion (51%) and rate (16.8 diagnoses per 100,000 population) of new diagnoses of HIV infection [2]. An antiretroviral medication, Truvada, is commonly used for pre-exposure prophylaxis (PrEP) and is highly effective in preventing HIV infections. PrEP treatment consists of daily use of tenofovir disoproxil fumarate (TDF) and a second medication, emtricitabine (FTC), in combination. Truvada is currently one of two medications approved by the US Food and Drug Administration (FDA) for PrEP. When used consistently and correctly by HIV-negative individuals at risk of acquiring HIV, medication-based PrEP has been shown to reduce the risk of acquisition of HIV infection through sex by more than 90% and by more than 70% among people who inject drugs based on studies that used TDF alone [3]. However, the use of these highly effective interventions by those who can most benefit from them has lagged [4]. Expanding access to PrEP to more individuals at high risk for acquiring HIV represents an important opportunity to help end the HIV epidemic in this country. The President’s Budget for fiscal year 2020 included a legislative proposal, “Ending the HIV Epidemic: A Plan for America,” which aims to reduce new infections by 75% by 2025 and by 90% by 2030 [5]. The proposal’s 4 key strategies are 1) diagnose all individuals with HIV as early as possible, 2) treat people with HIV rapidly and effectively to reach sustained viral suppression, 3) prevent new HIV transmissions by using proven interventions, including PrEP and syringe service programs, and 4) respond quickly to potential HIV outbreaks to get needed prevention and treatment services to people who need them [6]. For instance, Health Resources and Services Administration (HRSA) recently announced plans to devote Health Center resources to expand PrEP services to selected health centers in jurisdictions where over half of all new infections occur [7].

The Centers for Disease Control and Prevention (CDC) estimated that in 2015, 1.1 million HIV-negative adults had indications for PrEP and could have benefited from PrEP [8]. Prior studies have estimated the number of PrEP patients (9,375 with commercial insurance in 2014; 9,684 in 2015; 70,395 in 2017) [9,10,11]; however, little is known about PrEP uptake at the metropolitan statistical area (MSA) level [8,11], characteristics of prescribers, or patient and insurance payments for PrEP [12]. This information may help identify for whom and where to focus efforts to increase use of PrEP and shed light on the cost of the medication for payors and patients.

In this study, we examined a large, nationally representative prescription claims database to 1) compare the characteristics of patients who take PrEP (including age, race/ethnicity, geography, and sex) to individuals with newly diagnosed HIV infection, 2) identify the specialties of practitioners prescribing PrEP, 3) identify areas of the US at the MSA level where there is relatively low uptake of PrEP, and 4) report average amounts paid by patients and third-party payors for PrEP.

## Methods

### Data

This study is reported as per the Strengthening the Reporting of Observational Studies in Epidemiology (STROBE) guidelines (S1 Text). To identify PrEP prescriptions, we used patient-linked claims from September 2015 through August 2016 from the Integrated Dataverse (IDV) prescription claims database produced by Symphony Health. The IDV contains longitudinal data that capture adjudicated prescription, medical, and hospital claims across the US for all payment types, including commercial plans, Medicare Part D, cash, assistance programs, and Medicaid. The IDV contains over 10 billion deidentified prescriptions claims linked to over 280 million unique patients with an average of 5 years of prescription drug history. These prescription drug claims are linked to hospital and physician practices claims with medical procedure (i.e., CPT, HCPCS) and diagnosis codes (ICD- 9/10) for nearly 180 million patients. The full database includes claims from over 65,000 pharmacies, 1,500 hospitals, 800 outpatient facilities, and 80,000 physician practices across the US, capturing approximately 75% of the total prescriptions dispensed in the US. The distribution of Symphony Health patients across census regions is very similar to that of the US population according to the US Census Bureau (Table A in S1 Appendix). Although substantial in scope, the data from IDV represent a convenience sample of the overall universe of prescriptions in the US.

**Fig 1:**
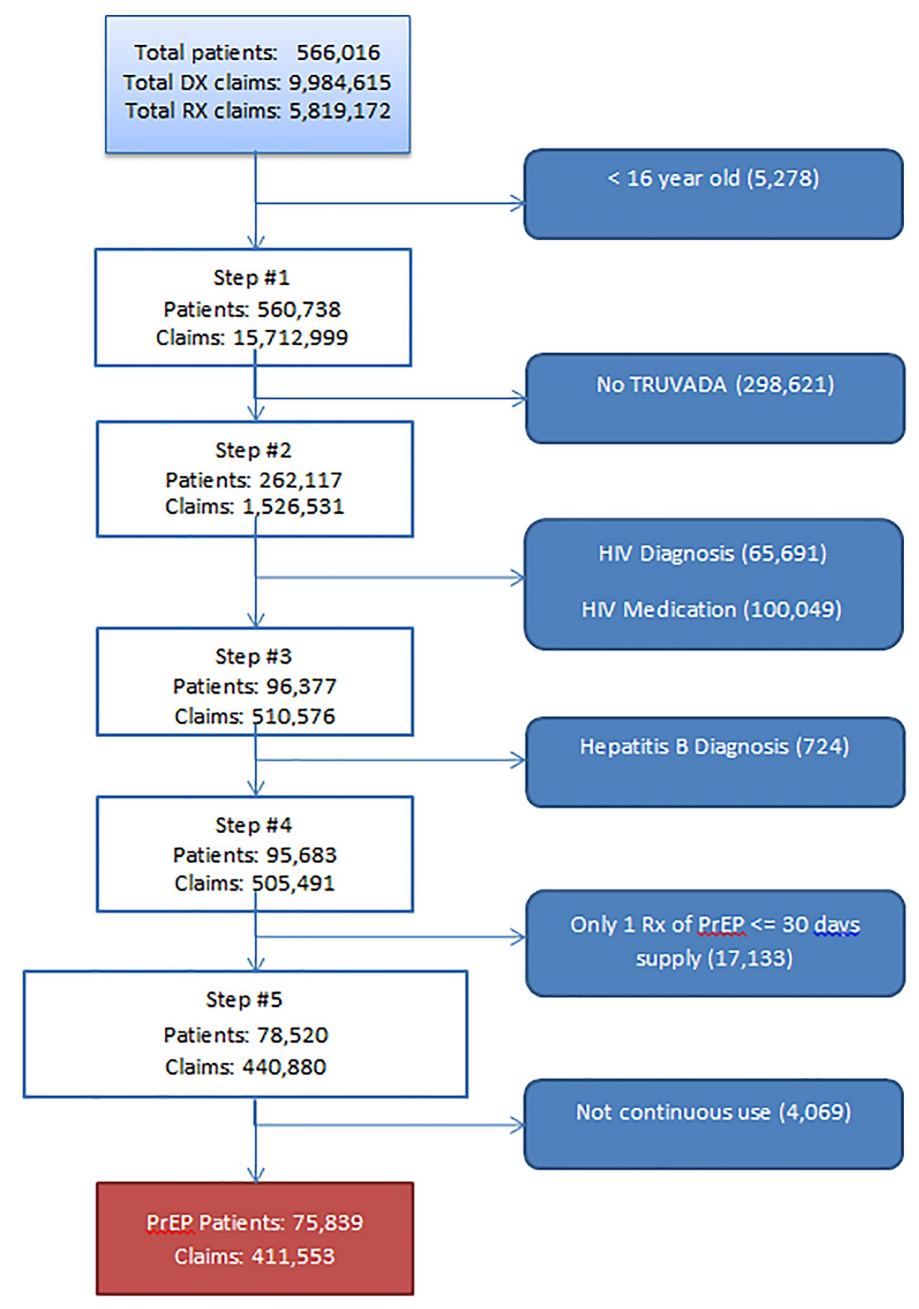
PrEP patient algorithm applied to IDV database, September 2015–August 2016. Dx, diagnosis; IDV, Integrated Dataverse; PrEP, pre-exposure prophylaxis; Rx, prescription.

To define our patient population, we first took a subset of the IDV database of only those patients with a prescription for the combination of FTC and TDF, or Truvada (TDF/FTC). We then excluded patients who used any of the antiviral drugs lamivudine, efavirenz, zidovudine, lopinavir/ritonavir, raltegravir, dolutegravir, darunavir, and ritonavir to remove patients using TDF/FTC and the aforementioned drugs as part of a drug regimen to treat HIV or hepatitis B. We followed the same algorithm used in Wu and colleagues [10] to identify claims for PrEP (Fig 1). An individual in the claims data was considered to be a PrEP patient if 16 years old or older with at least one prescription for TDF/FTC and without diagnosis codes or prescription claims of medication indicative of having HIV or hepatitis B infection. Lastly, we excluded individuals who had been prescribed TDF/FTC for 30 days or less, the same algorithm used in the indicator for the National HIV/AIDS Strategy for PrEP, and assumed that they either used TDF/FTC for postexposure prophylaxis (PEP) or that they were not taking PrEP effectively, as had been assumed in prior work [10]. The prespecified study plan is available in S2 Text. Following review of initial results, we carried out further non-prespecified analyses to 1) compare the characteristics of patients prescribed PrEP to individuals with diagnoses of new HIV infections, 2) use MSA as a more granular level of geography for patient characteristics, and 3) calculate payments for PrEP by payor type.

IDV data include geographic information for patient residence location at the US Census Bureau region level, whereas practitioner practice location is available at the ZIP code level. A patient can have multiple practitioners who prescribed PrEP, but we used only one practitioner and the practitioner’s ZIP code information per patient. We assigned the ZIP code of the physician who had the largest number of claims for PrEP for a given patient to that patient’s residence. We aggregated ZIP codes to the MSA level because this allowed us to compare the number of individuals prescribed PrEP to the number of individuals with newly diagnosed HIV infection, a number that is also available at the MSA level in CDC’s HIV Surveillance Report [13]. We compared the characteristics of PrEP patients in IDV with those of individuals with newly diagnosed HIV infection nationwide as reported in the CDC’s 2015 annual HIV Surveillance Report [13]. The latter represents individuals who might have avoided HIV infection had they been using PrEP. We also used these data to approximate relative levels of geographic uptake of PrEP by generating a ratio of the number of PrEP patients within an MSA (in the numerator) to the number of newly diagnosed individuals with HIV (in the denominator).

### Analysis

We calculated descriptive statistics at the national level for demographic characteristics available in the database (age, race and ethnicity, census region, sex, educational attainment, and household income) for PrEP patients and compared these statistics using a chi-squared test with the characteristics of diagnoses of new HIV infections found in CDC’s HIV Surveillance Special Report 2015 [14]. We also compared age, race and ethnicity, and sex at the MSA level between the 2 groups to find possible MSAs where there might similar or dissimilar demographic compositions using CDC’s HIV Surveillance Supplemental Report 2015 [15]. There were missing data in the IDV for demographic characteristics provided in the IDV database such as patient race and ethnicity (35.2% missing), household income (36.2%), and educational attainment (33.9%). The categories of missing or unknown were not included in the chi-squared test. To confirm whether this analysis using IDV data is consistent with previous work, we compared PrEP patients having commercial insurance in the IDV database to PrEP patients in a prior analysis using the 2010–2014 MarketScan Commercial Claims and Encounters database [10]. This comparison showed similar demographics for commercially insured PrEP patients in the IDV and MarketScan databases (Table B in S1 Appendix), except for geographic distribution.

We aggregated the number of PrEP patients by MSA (we only included 107 MSAs with populations of 500,000 or greater in our analysis), which we linked to the ZIP code information provided in the IDV database for the patient’s provider who prescribed PrEP, and reported the 20 MSAs with the lowest and the 20 MSAs with the highest relative use of PrEP. Relative use of PrEP is calculated using the ratio of PrEP patients (numerator) to the number of individuals newly diagnosed with HIV infection in 2015 (denominator).

To calculate the median, average, 25th and 75th percentile payments made by patients and third-party payors (commercial insurance, Medicaid [includes both fee-for-service and managed care], Medicare, TRICARE, Gilead discount program, and other assistance) and the standard deviation for these payments, we first totaled payments by payor type in each month for each patient. Both the patient and third-party payor categories were provided in the IDV database. There were a small number of cases of multiple payors within the same patient-month (1.2%). We were able to identify that a payment made by a secondary third-party payor covered the patient cost-sharing amount for the primary payment method (e.g., Gilead’s payment canceled the patient payment for commercial insurance). We averaged these payment amounts across all months of data available for each patient. These average payment amounts were then multiplied by 12 to annualize the data. Finally, we calculated these statistics across all patients by payor type using the annualized payment amounts. We annualized the data to avoid giving greater weight to patients with more months of data. For those without insurance and with household incomes less than 500% of the federal poverty level calculated in 2015, Gilead offers a medication assistance plan that provides free medication. Gilead also offers a payment assistance program to pay for health insurance copays up to US$3,600 annually. Because PrEP patients may appear in more than one category of third-party payor, these categories are not mutually exclusive. We compared the payment methods for PrEP purchases by PrEP patients with the types of payment methods used by people living with diagnosed HIV (PLWH) infection during 2015 and 2016 for antiretroviral medications in the past 12 months using Table 2 in the CDC HIV Surveillance Special Report 2015 [14].

All data analyses were calculated using SAS version 9.4 (SAS Institute Inc., Cary, NC, USA; 2016). Specific ethics approval was not required for this secondary data analysis.

## Results

### Characteristics of PrEP patients

A total of 75,839 individuals in the IDV database were prescribed PrEP during September 2015 through August 2016; this number assumes that the IDV database captures approximately 75% of the total prescriptions dispensed in the US. We then extrapolated to 100% of total prescriptions dispensed, assuming the same prevalence of prescription among those included in IDV as those excluded, and estimated that approximately 101,000 patients were prescribed PrEP nationally. There are estimated to be 1.1 million adults in the US with an indication for PrEP [8], suggesting that fewer than 10% of those who could potentially benefit from PrEP received the drug during the study period.

Comparing individuals prescribed PrEP in the IDV database to those with newly diagnosed HIV infection in 2015, PrEP patients were more likely to be non-Hispanic white (45% versus 26.2%), be older (25% versus 19% at ages 35–44), be male (94% versus 81%), and not reside in the South (30% versus 52% reside in the South) (Table 1). All differences were statistically significant using a chi-squared test with a *p*-value less than 0.001. Supplemental information on how PrEP patients differed from individuals with newly diagnosed HIV infection by age, race and ethnicity, and sex at the MSA level is provided in Table B in S1 Appendix. These findings may help identify populations where efforts to increase uptake of PrEP could be targeted.

**Table 1:** Demographic characteristics of individuals prescribed PrEP in IDV database and adults and adolescents diagnosed with HIV infection reported to the CDC in 2015.

		Number of Individuals Prescribed PrEP, September 2015–August 2016	%	Number of Individuals with Newly Diagnosed HIV Infection, 2015	%	*p*-Value***
Total		75,839	100.0	39,741	100.0	
Age^						<0.001
	13–14	Not Available		26	0.1	
	15–34	34,935	46.1	22,010	55.4	
	35–44	18,753	24.7	7,669	19.3	
	45–54	15,088	19.9	6,306	15.9	
	55–64	5,781	7.6	2,883	7.3	
	≥65	1,282	1.7	847	2.1	
Ethnicity						<0.001
	Black/African American	5,944	7.8	17,345	43.6	
	Hispanic/Latino	6,523	8.6	9,682	24.4	
	White/Caucasian	34,427	45.4	10,447	26.3	
	Other	2,230	2.9	2,267	5.7	
	Unknown	26,715	35.2	Not Available		
Geography—Census Region						<0.001
	Midwest	12,298	16.2	5,198	13.1	
	Northeast	20,243	26.7	6,478	16.3	
	South	22,550	29.7	20,377	51.3	
	West	20,132	26.6	7,688	19.3	
	Other/unknown	616	0.8	Not Available		
Sex						<0.001
	Male	71,349	94.1	32,306	81.3	
	Female	4,490	5.9	7,435	18.7	
Educational Attainment						N/A
	HS graduate or less	13,447	17.7	Not Available		
	Some college	16,790	22.1	Not Available		
	Associate degree/bachelor degree or more	19,885	26.2	Not Available		
	Unknown	25,717	33.9	Not Available		
Household Income						N/A
	Under 30,000	4,571	6.0	Not Available		
	30,000–49,000	8,158	10.8	Not Available		
	50,000–74,000	11,412	15.1	Not Available		
	75,000–99,000	8,704	11.5	Not Available		
	100,000+	15,523	20.5	Not Available		
	Unknown	27,471	36.2	Not Available		

Source: Authors’ analysis of the IDV data from September 2015 to August 2016.

***p-value for chi-squared tests.

^Age categories for PrEP patients are 16–35, 36–45, 46–55, 55–65, over 65.

**Abbreviations:** CDC, Centers for Disease Control and Prevention; IDV, Integrated Dataverse; N/A, not applicable; PrEP, pre-exposure prophylaxis.

### Uptake of PrEP by MSA

**Fig 2:**
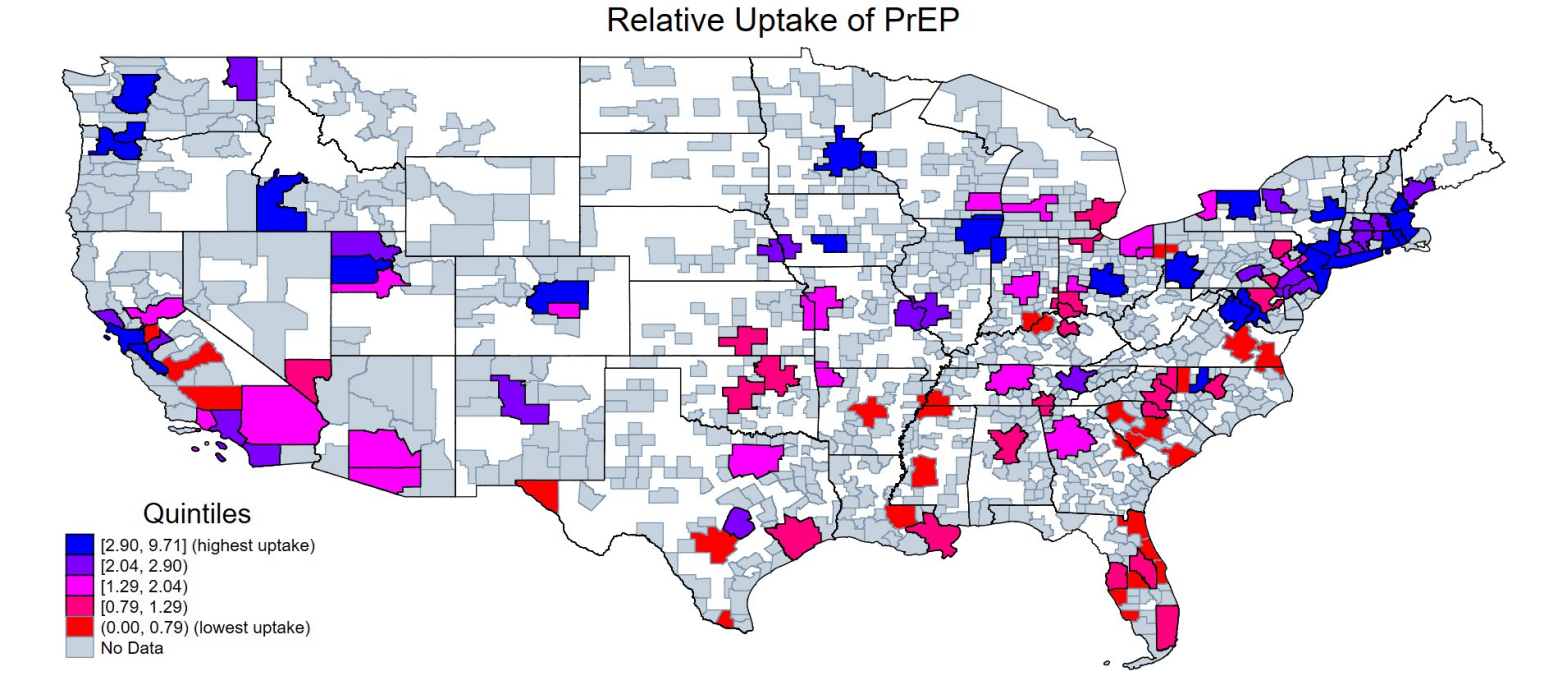
Relative uptake of PrEP by MSA for MSAs with populations of 500,000 or greater. Source: Authors’ analysis of the IDV data from September 2015 to August 2016 and CDC’s 2015 Annual HIV Surveillance Report. The authors used a shapefile, rather than a basemap, of the US with state borders from the US Census Bureau, https://www.census.gov/geographies/mapping-files/time-series/geo/tiger-line-file.html. CDC, Centers for Disease Control and Prevention; IDV, Integrated Dataverse; MSA, metropolitan statistical area; PrEP, pre-exposure prophylaxis.

The 20 MSAs ranked as having the lowest and highest amounts of uptake of PrEP are shown in Table 2 (see Table D in S1 Appendix for relative uptake for the full list of 107 MSAs). Fig 2 depicts this information geographically with relative uptake of PrEP color coded by quintiles, with red indicating MSAs with the lowest uptake of PrEP. The 3 MSAs with the lowest relative uptake of PrEP are McAllen–Edinburg–Mission, TX (ratio of 0.10), Virginia Beach–Norfolk–Newport News, VA–NC (0.15), and Baton Rouge, LA (0.18).

**Table 2:** Number of PrEP patients, number of newly diagnosed HIV infections in 2015, and ratio of relative uptake by MSA for the 20 MSAs with the lowest and highest uptake.

MSA	Number of PrEP Patients	Number of People with Newly Diagnosed HIV Infection, 2015	Ratio of Number of PrEP Patients to Number of Newly Diagnosed HIV Infections	Ranking of Ratio
Twenty MSAs by Lowest Uptake				Lowest 20 MSAs
McAllen–Edinburg–Mission, TX	9	82	0.11	1
Virginia Beach–Norfolk–Newport News, VA–NC	53	293	0.18	2
Baton Rouge, LA	54	265	0.20	3
Deltona–Daytona Beach–Ormond Beach, FL	22	78	0.28	4
San Juan–Carolina–Caguas, PR	144	399	0.36	5
Palm Bay–Melbourne–Titusville, FL	21	57	0.37	6
Augusta–Richmond County, GA–SC	41	104	0.39	7
Bakersfield, CA	49	121	0.40	8
Lakeland–Winter Haven, FL	43	106	0.41	9
Memphis, TN–MS–AR	127	312	0.41	10
Stockton–Lodi, CA	28	68	0.41	11
El Paso, TX	48	116	0.41	12
Jacksonville, FL	131	315	0.42	13
Youngstown–Warren–Boardman, OH–PA	15	35	0.43	14
Columbia, SC	81	164	0.49	15
Greenville–Anderson–Mauldin, SC	39	78	0.50	16
Greensboro–High Point, NC	67	131	0.51	17
San Antonio–New Braunfels, TX	200	386	0.52	18
Richmond, VA	135	227	0.59	19
Fresno, CA	61	102	0.60	20
Twenty MSAs by Highest Uptake				Highest 20 MSAs
Madison, WI	204	21	9.71	1
Seattle–Tacoma–Bellevue, WA	2,877	334	8.61	2
San Francisco–Oakland–Hayward, CA	5,625	722	7.79	3
Boston–Cambridge–Newton, MA–NH	2,876	456	6.31	4
Portland–Vancouver–Hillsboro, OR–WA	897	165	5.44	5
Des Moines–West Des Moines, IA	169	32	5.28	6
Salt Lake City, UT	384	75	5.12	7
Minneapolis–St. Paul–Bloomington, MN-WI	1,212	265	4.57	8
Albany–Schenectady–Troy, NY	223	50	4.46	9
Rochester, NY	375	87	4.31	10
Chicago–Naperville–Elgin, IL–IN–WI	5,347	1,380	3.87	11
Providence–Warwick, RI–MA	405	107	3.79	12
Pittsburgh, PA	622	165	3.77	13
Boise City, ID	72	20	3.60	14
New York–Jersey City, NY–NJ–PA	12,402	3,563	3.48	15
Durham–Chapel Hill, NC	279	81	3.44	16
Columbus, OH	826	240	3.44	17
San Jose–Sunnyvale–Santa Clara, CA	534	156	3.42	18
Washington–Arlington–Alexandria, DC–VA–MD–WV	3,941	1,233	3.20	19
Denver–Aurora–Lakewood, CO	789	272	2.90	20

Source: Authors’ analysis of the IDV data from September 2015 to August 2016 and the CDC’s 2015 annual HIV Surveillance Report. **Abbreviations:** CDC, Centers for Disease Control and Prevention; IDV, Integrated Dataverse; MSA, metropolitan statistical area; PrEP, pre-exposure prophylaxis.

### Specialties and locations of PrEP-prescribing practitioners

A total of 23,955 practitioners out of approximately 1.8 million prescribers in the IDV database prescribed PrEP. Although a variety of specialties were reported for these practitioners, two-thirds reported primary care (i.e., internal medicine, family medicine, and family practice) as their specialty (Table E in S1 Appendix; Table F in S1 Appendix for the top 20 diagnoses for PrEP patients; and Table G in S1 Appendix for the top 20 MSAs by number of PrEP-prescribing practitioners). Provider specialty is self-reported, and some of the categories may overlap (e.g., family medicine and family practice).

### Paying for PrEP

On average, individuals prescribed PrEP used 1.013 (range: 1 to 3) different payment methods to pay for PrEP each month. Payment methods included commercial health insurance, Medicaid, Medicare, TRICARE, Veterans Administration (VA), Gilead’s discount program, cash, and other assistance programs. Most (80%) PrEP patients in the IDV database used commercial health insurance, alone or in conjunction with other payment methods, to pay for PrEP (Table 3), followed by patients with Gilead’s assistance program (12.51%), Medicaid (11.83%), Medicare (4.25%), cash (3.76%), other assistance (2.20%), and TRICARE (0.56%). Patients can have multiple third-party payors. Compared to the payment methods used by PLWH in 2015 and 2016 for antiretroviral medications, PrEP patients were more likely to use commercial or private health insurance (80% for PrEP patients versus 35% for PLWH) and less likely to use public insurance or assistance programs.

**Table 3:** Payment methods of PrEP patients, September 2015 through August 2016, and type of health insurance of coverage for antiretroviral medications for PLWH.

Payment Methods	Number of PrEP Patients	Proportion of All PrEP Patients (%)	Proportion of Individuals Living with Diagnosed HIV (%)
Commercial	60,580	79.88%	34.90%
Medicaid	8,970	11.83%	44.80%
Medicare	3,224	4.25%	27.50%
TRICARE/CHAMPUS or VA (VA not included in PrEP patient data)	428	0.56%	4.80%
Gilead	9,487	12.51%	n/a
Cash	2,851	3.76%	n/a
Other assistance	1,672	2.20%	n/a
Ryan White	n/a	n/a	44.90%
Other public insurance	n/a	n/a	12.50%
Insurance type unknown	n/a	n/a	1.50%
No health insurance or coverage	n/a	n/a	1.90%

Source: Authors’ analysis of the IDV data from September 2015 to August 2016.

Each payment method is not mutually exclusive; i.e., patients may use more than one type of payment method and appear in more than one row in this table. The number of individuals prescribed PrEP in Table 3 adds up to more than 75,839. The percent of individuals prescribed PrEP does not sum to 100% for the same reason. There are very few patients who are dually eligible for Medicare and Medicaid. Gilead is parsed out from “Other assistance.” “TRICARE” is parsed out from commercial insurance. We identified 4 patients with payments for PrEP made by the VA and are not reporting on them due to the small sample size. **Abbreviations:** IDV, Integrated Dataverse; PLWH, people living with diagnosed HIV; PrEP, pre-exposure prophylaxis; VA, Veterans Administration.

The estimated median annual per patient spending on PrEP medication was US$72 in out-of-pocket spending for patients and US$17,496 across all third-party payors (see Table 4). Commercial health insurance plans covered approximately 98% (US$17,568 per patient per year) of the costs of TDF/FTC for their enrollees, a coverage amount similar to Medicaid (>99%), Medicare (>99%), TRICARE (99%), and Gilead for those who qualify (>99%). For patients with Medicare, the low median patient payment appears to be explained by the high percentage (67%) of Medicare beneficiaries in this patient population who receive the Low-Income Subsidy under the Medicare Part D prescription drug program. Approximately 15% of commercially insured PrEP patients had cost sharing equal to or exceeding US$3,697 per year (US$925 average yearly payment with commercial insurance; ±US$2,772 standard deviation [see Table H in S1 Appendix]), or US$308 per month.

**Table 4:** Median patient and TPP payments for PrEP by payment method category, September 2015 through August 2016.

Payment Method	Number of PrEP User-Months	Patient and TPP	Median Monthly Payment	25th Percentile Monthly Payment	75th Percentile Monthly Payment	TPP Share of Monthly Payment (Median)	Projected Yearly Payment (Median)
Any Insurance	264,929	Patient	$6	$0	$0		$72
		TPP	$1,458	$1,379	$1,498	>99%	$17,496
Commercial	187,148	Patient	$30	$0	$50		$360
		TPP	$1,464	$1,389	$1,509	98%	$17,568
Medicaid	30,580	Patient	$0	$0	$3		$0
		TPP	$1,468	$1,396	$1,483	>99%	$17,616
Medicare	13,487	Patient	$1	$0	$10	0%	$12
Low-Income Subsidy	10,185	Patient	$0	$0	$4	0%	$0
Other	3,302	Patient	$74	$50	$474	5%	$888
		TPP	$1,462	$1,379	$1,494	>99%	$17,544
Tricare	2,013	Patient	$20	$0	$24		$240
		TPP	$1,429	$1,358	$1,445	99%	$17,148
Gilead	27,737	Patient	$0	$0	$0		$0
		TPP	$75	$35	$1,480	>99%	$900
Cash Only	118	Patient	$1,791	$1,716	$1,945		$21,492
		TPP	$0	$0	$0	0%	$0
Other Assistance	3,281	Patient	$0	$0	$0		$0
		TPP	$1,399	$74	$1,510	>99%	$16,788

Source: Authors’ analysis of the IDV data from September 2015 to August 2016.

Notes: Each payment method category is not mutually exclusive, except for the category “Cash Only.” That is, patients may have more than one type of insurance and appear in more than one row in this table. The exception is “Cash Only”; patients in this category only paid for PrEP using cash. The number of individuals prescribed PrEP in Table 4 adds up to more than 75,839. The percent of individuals prescribed PrEP does not sum to 100% for the same reason. “TRICARE” is parsed out from commercial insurance. We identified 4 patients with payments for PrEP made by the VA and are not reporting on them due to the small sample size. There are very few patients in this database who are dually eligible for Medicare and Medicaid. **Abbreviations:** IDV, Integrated Dataverse; PrEP, pre-exposure prophylaxis; TPP, third-party payor; VA, Veterans Administration.

## Discussion

In this study of a US prescription claims database for the period of September 2015 to August 2016, we found that less than 10% of individuals indicated for PrEP were prescribed PrEP, suggesting that many people who might have benefited from PrEP were not receiving it. In 2016, Black/African American and Hispanic/Latino individuals accounted for the highest rates of diagnoses of HIV infection [2], but these groups accounted for low proportions (7.8% and 8.6%, respectively) of PrEP patients in our analysis. This disparity by race and ethnicity is consistent with previous research [8,16]. We found that MSAs with low relative uptake of PrEP tended to be concentrated in the South, which is also where the incidence of HIV is the highest in the country [2]. Additional efforts could be employed to increase uptake of PrEP in these populations and areas of the country. Some local and state government agencies have developed and implemented community outreach and social media campaigns that may contribute to increased awareness and uptake of PrEP [16]. In New York, e.g., the health department in 2015 featured advertisements in subway stations, on Metropolitan Transportation Authority (MTA) buses, and online [17]. Health departments in San Francisco, the District of Columbia, and other cities have also launched campaigns involving promotion of PrEP [18]. In Florida, the Department of Health planned to make PrEP available at no cost through county health departments by the end of 2018 [19]. Similar efforts could be undertaken in other areas of the South, given we found that PrEP uptake was relatively lower in the South.

We found that PrEP patients are more likely to pay for PrEP using commercial or private insurance, which helped substantially offset the cost of TDF/FTC (median out-of-pocket payments for patients with commercial insurance were US$30 per month), whereas patients living with HIV are more likely to pay for their antiretroviral treatment using public insurance (Medicaid and Medicare) or a publicly sponsored assistance program (Ryan White HIV/AIDS Program). For PLWH who are uninsured or underinsured, the Ryan White HIV/AIDS Program covers the costs of antiretroviral medication to treat HIV, among other HIV-related treatment services, as a secondary payer but cannot pay for PrEP medications for HIV-negative individuals. Given that many individuals receiving antiretroviral medication are paying for this treatment using payment sources other than private insurance, this indicates that many of those who could benefit from taking PrEP may not be able to afford PrEP, since many of them appear to not have private insurance coverage. Concerns related to cost and lack of insurance or underinsurance can be a barrier to use of PrEP [16]. One study seemed to counter this notion with its findings that between 50% to 75% of individuals indicated for PrEP have public or private insurance to cover most PrEP care costs [12]. We were unable to ascertain the number of individuals with private insurance that does not cover TDF/FTC or the number of individuals who may forgo TDF/FTC treatment because of the level of patient cost sharing. Regardless, even among those who have insurance that could cover the costs of PrEP, there may still be perceived concerns about the affordability of PrEP given its price. It is also possible that some individuals indicated for PrEP were not aware of the availability of the Gilead assistance program, which might have lowered the cost of the medication for some of these patients. In June 2019, the US Preventive Services Task Force (USPSTF) published a final recommendation statement on PrEP. The USPSTF recommends that clinicians offer PrEP to people at high risk of HIV acquisition, giving this recommendation an A grade [20]. Under current law, most private insurance plans are required to cover USPSTF Grade A and B recommendations with no out-of-pocket costs [21]. In order for this policy to affect uptake, it will be important for high-risk individuals to be become aware that this is the case.

From a third-party payor perspective, given our estimate of the median annual cost of PrEP to third-party payors (US$72 + U$17,496 = US$17,568), PrEP costs approximately 36% less than the annual per-person cost of HIV treatment and care, previously estimated at US$27,461 (2016 US dollars) [22,23]. This comparison only includes the cost of medication for PrEP and not the costs of other services associated with taking PrEP, including follow-up office visits and laboratory tests that should be conducted every 3 months to screen for HIV, STDs, pregnancy for women who may become pregnant, and potential effects of the medication on kidneys [3]. However, even when including clinical costs, another study found PrEP to be highly cost-effective for high-risk populations [24]. In June 2017, the US FDA approved production of generic Truvada by Teva Pharmaceuticals as a component of an HIV treatment regimen and as PrEP [25]. Generic Truvada is still not available in the US market [25], but Gilead Sciences announced that Teva Pharmaceuticals will be able to launch a generic versions of Truvada in the US on September 30, 2020 [26,27]. When the generic formulation becomes available in the US, it is possible that the price for this drug may decrease, but generic manufacturers often hold exclusive rights for an initial period before competitors can also begin producing the medication and drive down prices [25]. In Europe, several generic manufacturers have received marketing approval from the European Medicines Agency for tenofovir disoproxil with emtricitabine (TDX/FTC), a bioequivalent to TDF/FTC [28].

Other barriers, beyond financial access, to uptake include low awareness of PrEP among potential patients [29,30,31] and their practitioners [32,33,34]. Primary care providers most frequently identified limited knowledge of PrEP and concerns regarding insurance coverage as prescribing barriers [32]. Mistrust of the medical system and perceived discrimination can also create barriers to accessing PrEP and other forms of HIV prevention [35,36].

Our study has some limitations and required several assumptions. First, we used claims data for this analysis, which exclude uninsured individuals who acquired PrEP purely through out-of-pocket spending. Given the cost of PrEP, this population would have likely had more financial resources relative to those not acquiring PrEP. The extrapolated number of PrEP patients assumes that the percentage of PrEP claims in the IDV database out of all possible drug claims in the US is the same as the average percentage across all drugs in the database out of all possible drug claims in the US (75%). Approximately 41.6% of the individuals identified in the subset of the IDV database have prescription drug claims but are missing diagnosis claims; we treated these individuals as not having HIV and/or hepatitis B. However, we were able to exclude nearly everyone with HIV by excluding patients with non-Truvada antiretroviral medication. Information on race and ethnicity, educational attainment, and income were missing for 34%–36% (depending on the variable) of PrEP patients in IDV. Although guidelines only recommend PrEP for patients 18 years or older, we included patients 16 years or older because age was provided as a categorical variable in the data we received (for privacy purposes), with the first relevant age category being 16 to 20 years old. We used 1 year of data to identify PrEP patients, but it is possible that a patient may have had an HIV or hepatitis B diagnosis in prior years, which would misattribute some number of people with HIV and/or hepatitis B infection as PrEP users. We used the number of newly diagnosed HIV cases in the denominator, which may be affected by the regional variation in the rate of diagnostic testing and delays between HIV infection and diagnosis [37]. We also assumed that the demographic and geographic characteristics of individuals with newly diagnosed HIV infection were similar to those at risk for HIV infection (i.e., people indicated for PrEP) because those with diagnosed with HIV were likely those who were most at risk of HIV infection given they actually acquired HIV. Ideally, we would have used estimates of the number of individuals at risk of HIV infection by MSA in 2016 in the denominator, but no known estimates exist. Given that we do not have access to medical claims in IDV, we were not able determine the length of time individuals may have been living with HIV prior to receiving a diagnosis. Finally, cases of HIV infection by victims of rape or assault, which represent a relatively small proportion of all individuals who acquire HIV [38], are unlikely to affect our estimates of relative uptake of PrEP.

We identified nearly 101,000 individuals prescribed PrEP during September 2015 through August 2016, which is fewer than 1 in 10 of those indicated for PrEP. Compared to individuals newly diagnosed with HIV in 2015, PrEP patients were more likely to be male, be non-Hispanic white, be older, have commercial insurance, and live in regions other than the South. Many individuals who may benefit from being on PrEP are not currently receiving this HIV prevention medication, and those prescribed PrEP have a significantly different distribution of characteristics from the broader population that is at risk for acquiring HIV. Median monthly patient cost sharing varied from US$0 for Medicaid beneficiaries to US$1,791 for those paying with cash.

Our findings indicate that many individuals who could potentially benefit from being on PrEP are not currently receiving this medication. Addressing the affordability of PrEP and otherwise promoting its use among those with indications for PrEP represents an important opportunity to help end the HIV epidemic.

## Supporting information

S1 Text Completed STROBE checklist. STROBE, Strengthening the Reporting of Observational Studies in Epidemiology.
(DOCX)

S2 Text Prespecified analysis plan. (DOCX)

S1 Appendix One file of supporting information in addition to the completed STROBE checklist. This files contains the following: a) Table A, IDV prescription patient distribution by census region; b) Table B, demographic characteristics of individuals prescribed PrEP with commercial health insurance from September 2015–August 2016 from IDV data and individuals prescribed PrEP with commercial health insurance in 2014 from MarketScan Data; c) Table C, summary of chi-squared test results comparing the age, race/ethnicity, and sex composition between PrEP patients and HIV diagnosis; d) Table D, number of PrEP patients, number of diagnoses of HIV infection in 2015, and ratio of relative uptake of PrEP by MSA, ranked by ratio from highest to lowest for 107 MSAs; e) Table E, number of practitioners prescribing PrEP by specialty, September 2015–August 2016; f) Table F, top 20 diagnoses of PrEP patients; g) Table G, top 20 MSAs by number of PrEP-prescribing practitioners, September 2015–August 2016; h) Table H, average patient and third-party payor payments for PrEP by payment method category, September 2015–August 2016. IDV, Integrated Dataverse; MSA, metropolitan statistical area; PrEP, pre-exposure prophylaxis; STROBE, Strengthening the Reporting of Observational Studies in Epidemiology.
(DOCX)

## Abbreviations

CDC: Centers for Disease Control and Prevention
FDA: Food and Drug Administration
FTC: emtricitabine
HHS: Health and Human Services
HRSA: Health Resources and Services Administration
IDV: Integrated Dataverse
MSA: metropolitan statistical area
MTA: Metropolitan Transportation Authority
PEP: postexposure prophylaxis
PLWH: people living with diagnosed HIV
PrEP: pre-exposure prophylaxis
STROBE: Strengthening the Reporting of Observational Studies in Epidemiology
TDF: tenofovir disoproxil fumarate
TDX/FTC: tenofovir disoproxil with FTC
USPSTF: US Preventive Services Task Force
VA: Veterans Administration
