# Supplementary material for: Pre-exposure prophylaxis for preventing acquisition of HIV: A cross-sectional study of patients, prescribers, uptake, and spending in the United States, 2015–2016
Source: PLoS Med. 2020 Apr 10;17(4):e1003072. doi: 10.1371/journal.pmed.1003072 (PMC7147726; doi:10.1371/journal.pmed.1003072)
Supplement: S1 Text — STROBE, Strengthening the Reporting of Observational Studies in Epidemiology. (DOCX) [file pmed.1003072.s001.docx]

**STROBE Statement—Checklist of items that should be included in reports of *cross-sectional studies***

**Manuscript title: Pre-exposure prophylaxis (PrEP) for preventing acquisition of HIV: A cross-sectional study of patients, prescribers, uptake, and spending in the United States, 2015-2016**

**Journal: PLOS Medicine (HRSA Supplement)**

Last updated: February 4, 2020

|  | Item No | Recommendation | Page No |
| --- | --- | --- | --- |
| **Title and abstract** | 1 | (*a*) Indicate the study’s design with a commonly used term in the title or the abstract  Cross-sectional descriptive study | Title Page |
|  |  | (*b*) Provide in the abstract an informative and balanced summary of what was done and what was found  Our findings indicate that, in 2015-16, many individuals in the US who could benefit from being on PrEP were not receiving this HIV prevention medication, and those prescribed PrEP had a significantly different distribution of characteristics from the broader population that is at risk for acquiring HIV. PrEP patients were more likely to pay for PrEP using commercial or private insurance, whereas PLWH were more likely to pay for their antiretroviral treatment using public insurance or a publicly-sponsored assistance program. | Abstract |
| Introduction | | | |
| Background/rationale | 2 | Explain the scientific background and rationale for the investigation being reported  The Centers for Disease Control and Prevention (CDC) estimated that in 2015, 1.1 million HIV-negative adults had indications for PrEP and could have benefited from PrEP. Prior studies have estimated the number of PrEP patients (9,375 with commercial insurance in 2014; 9,684 in 2015; 70,395 in 2017) ; however, little is known about PrEP uptake at the MSA level , characteristics of prescribers, or patient and insurance payments for PrEP. This information may help identify for whom and where to focus efforts to increase use of PrEP, and shed light on the cost of the medication for payors and patients. | Introduction, paragraph 2 |
| Objectives | 3 | State specific objectives, including any prespecified hypotheses  In this study, we examined a large, nationally representative prescription claims database to (1) compare the characteristics of patients who take PrEP, to individuals with newly diagnosed HIV infection; (2) identify the specialties of practitioners prescribing PrEP; (3) identify areas of the United States at the MSA-level where there is relatively low uptake of PrEP; and (4) report average amounts paid by patients and third-party payors for PrEP. | Introduction, last paragraph |
| Methods | | | |
| Study design | 4 | Present key elements of study design early in the paper  To identify PrEP prescriptions, we used patient-linked claims from September 2015 through August 2016 from the IDV® (Integrated Dataverse) prescription claims database produced by Symphony Health.  We also used these data to approximate relative levels of geographic uptake of PrEP by generating a ratio of the number of PrEP patients within an MSA (in the numerator) to the number of newly diagnosed individuals with HIV (in the denominator). The ratio can be interpreted as PrEP uptake relative to HIV risk, and Aa higher ratio indicates greater relative uptake of PrEP. | Methods, Data and Analysis sub-sections |
| Setting | 5 | Describe the setting, locations, and relevant dates, including periods of recruitment, exposure, follow-up, and data collection  To identify PrEP prescriptions, we used patient-linked claims from September 2015 through August 2016 from the IDV® (Integrated Dataverse) prescription claims database produced by Symphony Health.  The full database includes claims from over 54,000 pharmacies, 1,500 hospitals, 800 outpatient facilities, and 80,000 physician practices across the U.S., capturing approximately 75% of the total prescriptions dispensed in the United States. | 8, Methods, Data, paragraph 1 |
| Participants | 6 | (*a*) Give the eligibility criteria, and the sources and methods of selection of participants  To define our patient population, we first took a subset of the IDV database of only those patients with a prescription for the combination of emtricitabine (FTC) and tenofovir disoproxil fumarate (TDF), or Truvada (TDF/FTC). We then excluded patients who used any of the following antiviral drugs: lamivudine, efavirenz, zidovudine, lopinavir/ritonavir, raltegravir, dolutegravir, darunavir, and ritonavir, to remove patients using TDF/FTC and the aforementioned drugs as part of a drug regimen to treat HIV or hepatitis B. We followed the same algorithm used in Wu et al. to identify claims for PrEP (Figure 1). An individual in the claims data was considered to be a PrEP patient if 16 years old or older with at least one prescription for TDF/FTC, and without diagnosis codes or prescription claims of medication indicative of having HIV or hepatitis B infection. Lastly, we excluded individuals who had been prescribed TDF/FTC for 30 days or less, the same algorithm used in the indicator for the National HIV/AIDS Strategy for PrEP, and assumed that they either used TDF/FTC for post-exposure prophylaxis (PEP) or that they were not taking PrEP effectively, as had been assumed in prior work . | Methods, Data, paragraph 2 |
| Variables | 7 | Clearly define all outcomes, exposures, predictors, potential confounders, and effect modifiers. Give diagnostic criteria, if applicable  We calculated descriptive statistics at the national level for demographic characteristics (age, race and ethnicity, census region, sex, educational attainment, and household income – variables provided in the database) for PrEP patients and compared these statistics using a chi-square test to the characteristics of diagnoses of new HIV infections found in CDC’s 2015 HIV Surveillance Report.  We aggregated the number of PrEP patients by MSA (we only included 107 MSAs with populations of 500,000 or greater in our analysis), which we linked to the zip code information provided in the IDV database, and reported the 20 MSAs with the lowest and the 20 MSAs with the highest relative use of PrEP. Relative use of PrEP is calculated using This is the ratio of PrEP patients (numerator) to the number of individuals newly diagnosed with HIV infection in 2015 plus the number of PrEP patients in these MSAs (denominator).  To calculate the average, minimum, and maximum payments made by patients and third-party payors (commercial insurance, Medicaid [includes both fee-for-service and managed care], Medicare, TRICARE, Gilead discount program, and other assistance) and the standard deviation for these payments, we first totaled payments by payor type in each month for each patient. Both the patient and third-party payor categories were provided in the IDV database. | Methods, Analysis sub-section |
| Data sources/ measurement | 8* | For each variable of interest, give sources of data and details of methods of assessment (measurement). Describe comparability of assessment methods if there is more than one group  To identify PrEP prescriptions, patient characteristics, and payment sources we used patient-linked claims from September 2015 through August 2016 from the IDV (Integrated Dataverse) prescription claims database produced by Symphony Health.  We compared the characteristics of PrEP patients in IDV to those of individuals with newly diagnosed HIV infection nationwide as reported in the CDC’s 2015 annual HIV Surveillance Report . | Methods, Data sub-section |
| Bias | 9 | Describe any efforts to address potential sources of bias  We calculated statistics on the above characteristics across all patients by payor type using the annualized payment amounts. We annualized the data to avoid giving greater weight to patients with more months of data. | Methods, Analysis, paragraph 3  Discussion, paragraph 10 |
| Study size | 10 | Explain how the study size was arrived at  We included all individuals that we identified as have been prescribed PrEP, as described above for ‘Participants’.  Also see Figure 1 (page 10) | Methods, Data, paragraph 2 |
| Quantitative variables | 11 | Explain how quantitative variables were handled in the analyses. If applicable, describe which groupings were chosen and why  Demographic variables:  For the demographic variables, we simply used the categorical variables of age, race/ethnicity, sex, income, and educational attainment that were available in the IDV database without additional manipulation.  Measure of PrEP uptake:  We aggregated the number of PrEP patients by MSA (we only included 107 MSAs with populations of 500,000 or greater in our analysis), which we linked to the zip code information provided in the IDV database, and reported the 20 MSAs with the lowest and the 20 MSAs with the highest relative use of PrEP. Relative use of PrEP is calculated using This is the ratio of PrEP patients (numerator) to the number of individuals newly diagnosed with HIV infection in 2015 plus the number of PrEP patients in these MSAs (denominator).  Payment amounts:  To calculate the average, minimum, and maximum payments made by patients and third-party payors (commercial insurance, Medicaid [includes both fee-for-service and managed care], Medicare, TRICARE, Gilead discount program, and other assistance) and the standard deviation for these payments, we first totaled payments by payor type in each month for each patient. Both the patient and third-party payor categories were provided in the IDV database. There were cases of multiple payors within the same patient-month, and in some cases we were able to identify the payment from the third-party payor (TPP) of one payment method covered the patient payment for another payment method (e.g., Gilead’s TPP payment canceled the patient payment for Commercial Insurance). We averaged these payment amounts across all months of data available for each patient. These average payment amounts were then multiplied by 12 to annualize the data. Finally, we calculated these statistics across all patients by payor type using the annualized payment amounts. We annualized the data to avoid giving greater weight to patients with more months of data. For those without insurance and with household incomes less than 500% of the federal poverty level calculated in 2015, Gilead offers a medication assistance plan which provides free medication. Gilead also offers a payment assistance program to pay for health insurance co-pays up to $3,600 annually. Because PrEP patients may appear in more than one category of third-party payor, these categories are not mutually exclusive. We compared the payment methods for PrEP purchases by PrEP patients, to the types of payment methods used by persons living with diagnosed HIV infection during 2015 and 2016 for antiretroviral medications in the past twelve months using Table 2 in the CDC HIV Surveillance Special Report 2015 . | Methods, Analysis, paragraphs 2 and 3 |
| Statistical methods | 12 | (*a*) Describe all statistical methods, including those used to control for confounding  N/A | N/A |
|  |  | (*b*) Describe any methods used to examine subgroups and interactions  N/A | N/A |
|  |  | (*c*) Explain how missing data were addressed  There were missing data in the IDV for demographic characteristics provided in the IDV database such as patient race and ethnicity (35.2% missing), household income (36.2%), and educational attainment (33.9%). The categories of missing or unknown were not included in the chi-square test.  We considered using multiple imputation to impute the missing data, but decided against it mainly because doing so would essential mean creating values for the outcome variable. This study was interested in the demographics of PrEP patients and comparing them to newly diagnosed HIV infections. The distribution of these demographic variables was the very thing we aimed to find out, and imputing these values would distort that estimate. The remaining variables without information were Age and Sex, and we felt that using only two variables may be insufficient to impute the missing information for the three variables (race, income, education).  Approximately 41.6% of the individuals identified in the subset of the IDV database have prescription drug claims but are missing diagnosis claims; we treated these individuals as not having HIV and/or hepatitis B. | Method, Analysis, paragraph 1  Discussion, paragraph 9 |
|  |  | (*d*) If applicable, describe analytical methods taking account of sampling strategy  N/A | N/A |
|  |  | (*e*) Describe any sensitivity analyses  N/A | N/A |
| Results | | | |
| Participants | 13* | (a) Report numbers of individuals at each stage of study—eg numbers potentially eligible, examined for eligibility, confirmed eligible, included in the study, completing follow-up, and analysed  See Figure 1  To define our patient population, we first took a subset of the IDV®IDV database of only those patients with a prescription for the combination of emtricitabine (FTC) and tenofovir disoproxil fumarate (TDF), or Truvada (TDF/FTC). We then excluded patients who used any of the following antiviral drugs: lamivudine, efavirenz, zidovudine, lopinavir/ritonavir, raltegravir, dolutegravir, darunavir, and ritonavir, to remove patients using TDF/FTC and the aforementioned drugs as part of a drug regimen to treat HIV or hepatitis B.  This subset started with 566,016 patients. After applying criteria for indication for PrEP, there were 75,839 patients prescribed PrEP | Methods, Data, Figure 1 |
|  |  | (b) Give reasons for non-participation at each stage  See Figure 1  Reasons for exclusion from database: under 16 years old, no prescription of Truvada, HIV diagnosis or medication, Hepatitis B diagnosis, only one prescription of Truvada, and not continuous use. | Methods, Data, Figure 1 |
|  |  | (c) Consider use of a flow diagram  See Figure 1 | Methods, Data, Figure 1 |
| Descriptive data | 14* | (a) Give characteristics of study participants (eg demographic, clinical, social) and information on exposures and potential confounders  Comparing individuals prescribed PrEP in the IDV database to those with newly diagnosed HIV infection in 2015, PrEP patients were more likely to be non-Hispanic White (45% vs 26.2%), older (25% vs. 19% at age 35-44), male (94% vs. 81%), and not reside in the South (30% vs. 52% reside in the South) (Table 1). | Results, Characteristics of PrEP Patients, paragraph 2 |
|  |  | (b) Indicate number of participants with missing data for each variable of interest  Unknown Race/Ethnicity: 26,715  Unknown Census Region: 616  Unknown Education: 25,717  Unknown Household Income: 27,471 | Results, Characteristics of PrEP Patients, Table 1 |
| Outcome data | 15* | Report numbers of outcome events or summary measures  N/A | N/A |
| Main results | 16 | (*a*) Give unadjusted estimates and, if applicable, confounder-adjusted estimates and their precision (eg, 95% confidence interval). Make clear which confounders were adjusted for and why they were included  Unadjusted estimates:  Comparing individuals prescribed PrEP in the IDV database to those with newly diagnosed HIV infection in 2015, PrEP patients were more likely to be non-Hispanic White (45% vs 26.2%), older(25% vs. 19% at age 35-44), male (94% vs. 81%), and not reside in the South (30% vs. 52% reside in the South) (Table 1).  Most (80%) PrEP patients in the IDV database used commercial health insurance, alone or in conjunction with other payment methods, to pay for PrEP (Table 3); followed by patients with Gilead’s assistance program (12.51%), Medicaid (11.83%), Medicare (4.25%), Cash (3.76%), Other Assistance (2.20%), and TRICARE (0.56%). Patients can have multiple third-party payors. Compared to the payment methods used by PLWH in 2015 and 2016 for antiretroviral medications, PrEP patients were more likely to use commercial or private health insurance (80% for PrEP patients vs. 35% for persons living with diagnosed HIV) and less likely to use public insurance or assistance programs.  Estimated annual per patient spending on PrEP medication averaged $791 in out-of-pocket spending for patients and $20,017 across all third-party payors (Table 4; Appendix: Table A7 for patients who used only one payment type per month; Appendix: Table A6 for payments made by mutually exclusive payment methods). Commercial health insurance plans covered approximately 96% ($22,411 per patient per year) of the costs of TDF/FTC for their enrollees, a coverage amount similar to Medicare (96%), TRICARE (99%), and Gilead for those who qualify (97%). Approximately 15% of commercially insured PrEP patients had cost sharing equal to or exceeding $3,692 per year ($925 average yearly payment with commercial insurance; +/- $2,768 standard deviation), or $308 per month. Individuals with Medicaid coverage had the least amount of patient cost sharing, paying approximately $55 per year for PrEP medication.  Adjusted estimates:  N/A | Results  Characteristics of PrEP patients, paragraphs 2;  Paying for PrEP, paragraphs 1,2 |
|  |  | (*b*) Report category boundaries when continuous variables were categorized  N/A | N/A |
|  |  | (*c*) If relevant, consider translating estimates of relative risk into absolute risk for a meaningful time period  N/A | N/A |
| Other analyses | 17 | Report other analyses done—eg analyses of subgroups and interactions, and sensitivity analyses  N/A | N/A |
| Discussion | | | |
| Key results | 18 | Summarise key results with reference to study objectives  In this study of a prescription claims database for the period of September 2015 and August 2016, we found that given fewer than 10% of the individuals indicated for PrEP were actually prescribed PrEP, it appears that many individuals who might have benefited from being on PrEP were not receiving the medication. In 2016, Black/African Americans and Hispanic/Latino individuals accounted for the highest rates of diagnoses of HIV infection , but these groups accounted for low proportions (7.8% and 8.6%, respectively) of PrEP patients in our analysis. This disparity by race and ethnicity is consistent with previous research. , and additional efforts could be employed to increase uptake of PrEP in these populations. | Discussion, paragraph 1 |
| Limitations | 19 | Discuss limitations of the study, taking into account sources of potential bias or imprecision. Discuss both direction and magnitude of any potential bias  We used claims data for this analysis, which excludes uninsured individuals who may have acquired PrEP purely through out-of-pocket spending. Given the cost of PrEP, this population would have likely had more financial resources relative to those not acquiring PrEP.  The extrapolated number of PrEP patients assumes that the percentage of PrEP claims in the IDV database is the same as the average percentage across all drugs in the database and that the percentage of all drug claims in the database is the same for urban areas as it is for all geographic areas included in the database.  Approximately 41.6% of the individuals identified in the subset of the IDV database have prescription drug claims but are missing diagnosis claims; we treated these individuals as not having HIV and/or hepatitis B. However, we were able to exclude nearly everyone with HIV by excluding patients with non-Truvada anti-retroviral medication. Information on race and ethnicity, educational attainment, and income were missing for 34-36% (depending on the variable) of PrEP patients in IDV. Although guidelines only recommend PrEP for patients 18 years or older, we included patients 16 years or older, because age was provided as a categorical variable in the data we received (for privacy purposes), with the first relevant age category being 16 to 20 years old. We used one year of data to identify PrEP patients, but it is possible that a patient may have had an HIV or hepatitis B diagnosis in prior years, which would misattribute some number of people with HIV and/or hepatitis B infection as PrEP users. We used the number of newly diagnosed HIV cases in the denominator, which may be affected by the regional variation in the rate of diagnostic testing and delays between HIV infection and diagnosis . Ideally, we would have used estimates of the number of individuals at risk of HIV infection by MSA in 2016 in the denominator, but no known estimates exist. However, MSAs typically represent the largest population centers within states, and our denominator is highly correlated with the number of individuals with an indication for PrEP (R>0.95) at the state level. Given that we do not have access to medical claims in IDV, we were not able determine the length of time individuals may have been living with HIV prior to receiving a diagnosis. Finally, cases of HIV infection by victims of rape or assault, which represent a relatively small proportion of all individuals who acquire HIV , are unlikely to affect our estimates of relative uptake of PrEP. | Discussion, paragraphs 10 and 11 |
| Interpretation | 20 | Give a cautious overall interpretation of results considering objectives, limitations, multiplicity of analyses, results from similar studies, and other relevant evidence  All reporting and discussion of results were thoughtfully crafted and incorporates a cautious overall interpretation.  We found that 1) fewer than 10% of the individuals indicated for PrEP were actually prescribed PrEP, 2) the two race/ethnicity groups that accounted for the highest rates of diagnoses of HIV infection have the lowest use of PrEP, and 3) MSAs with low relative uptakes tended to be concentrated in the South. It appears that many individuals who might have benefited from being on PrEP were not receiving the medication, and additional efforts could be employed to increase uptake of PrEP in these populations and areas of the country. Some local and state government agencies have developed and implemented community outreach and social media campaigns that may contribute to increased awareness and uptake of PrEP. Similar efforts could be undertaken in other areas of the South, given we found that PrEP uptake was relatively lower in the South.  We found that PrEP patients are more likely to pay for PrEP using commercial or private insurance, which helped substantially offset the cost of TDF/FTC (the median out-of-pocket payments for insured patients was $6 per month, and commercial insurance’s share of the total payment was 98%). Even among those who have insurance that could cover the costs of PrEP, there may still be perceived concerns about the affordability of PrEP given its price. It is also possible that some individuals indicated for PrEP were not aware of the availability of the Gilead assistance program, which might have lowered the cost of the medication for some of these patients. | Results, paragraphs 1, 2  Discussion, paragraph 2 |
| Generalisability | 21 | Discuss the generalisability (external validity) of the study results  Data sources were collected for the US nationwide. Although substantial in scope, the data from IDV represents a convenience sample of the overall universe of prescriptions in the United States. | Methods, paragraph 1 |
| Other information | | | |
| Funding | 22 | Give the source of funding and the role of the funders for the present study and, if applicable, for the original study on which the present article is based  All co-authors have no financial disclosure to report. The data were purchased using federal government funds and all authors are federal employees. | N/A |

*Give information separately for exposed and unexposed groups.

**Note:** An Explanation and Elaboration article discusses each checklist item and gives methodological background and published examples of transparent reporting. The STROBE checklist is best used in conjunction with this article (freely available on the Web sites of PLoS Medicine at http://www.plosmedicine.org/, Annals of Internal Medicine at http://www.annals.org/, and Epidemiology at http://www.epidem.com/). Information on the STROBE Initiative is available at www.strobe-statement.org.
