# Supplementary material for: Pre-exposure prophylaxis for preventing acquisition of HIV: A cross-sectional study of patients, prescribers, uptake, and spending in the United States, 2015–2016
Source: PLoS Med. 2020 Apr 10;17(4):e1003072. doi: 10.1371/journal.pmed.1003072 (PMC7147726; doi:10.1371/journal.pmed.1003072)
Supplement: S2 Text — (DOCX) [file pmed.1003072.s002.docx]

# ANALYTIC WORKPLAN FOR PROJECT TITLE PrEP and nPEP: Investigating Geographic Variation in Utilization Trends

Last Updated: March 22, 2017

## Key Staff:

Stephanie Chan

Andre Chapel

Nguyen Nguyen

## Objective of the Project

Although HIV infection is preventable, there are around 50,000 new HIV infections each year in the U.S. Uninfected individuals at high risk of acquiring HIV through sex can substantially reduce their risk by taking oral antiretroviral **pre-exposure** prophylaxis (PrEP) via Truvada. In 2012, the FDA approved the use of the prescription drug Truvada, a combination of two medicines in one pill. Truvada for PrEP is intended for high risk individuals including 1) HIV-negative men who have sex with men who are at high risk of getting infected with HIV through sex, and 2) male-female sex partners when one partner has HIV infection and the other does not. In 2014, the U.S. Public Health Service issued clinical guidelines for the use of PrEP as one HIV prevention option “in combination with safer sex practices.” For individuals not receiving PrEP but who seek care within 72 hours after an isolated sexual or injection-related HIV exposure, non-occupational **post-exposure** prophylaxis (nPEP) is recommended to decrease their risk of seroconversion, or the interval of several weeks after HIV infection when the body produces detectable levels of HIV antibodies.

There are other studies about Truvada use but there is little known about utilization by geographic areas or provider characteristics.

This project will provide HHS with a better understanding of Pre- and Post-Exposures Prophylaxis (PrEP and PEP) utilization as it relates to identified high HIV prevalence areas. Identifying geographic variation in the utilization of these HIV risk reduction strategies would help identify locales where greater investment in provider education may be warranted to increase PrEP and PEP utilization.

## Policy Relevance

This work is aligned with the HHS Strategic Goal 3: Advance the Health, Safety, and Well-Being of the American People to:

- Promote prevention and wellness across the life span.
- Reduce the occurrence of infectious diseases.

## Research Questions

1. What are the utilization rates of PrEP and nPEP in the U.S. for the most recent calendar year?
2. What is the degree of utilization of PrEP by geography and when considering doctor and patient demographics, including insurance coverage, age, and gender?

## Data

### Symphony Health Solutions

Prescription claims data for those prescribed PrEP/nPEP at any time from September 2015 to August 2016 in the Symphony Health Solutions database, with the below “Patient Qualifications”.

The dataset includes information on: patient level demographics, prescription drug activity (date prescription was filled, days of supply, number of refills authorized), drug description (e.g., drug name, drug strength), insurance plans, physician characteristics, diagnosis and procedure codes. The data include the address, city, state, and zip code for the prescribing physician. The prescription data will be summarized at the physician level, and they will provide patient age group, gender, education level, income level, ethnicity, and Census region of residence.

Patient Qualifications include any patients who have used the following drug utilization:

- Lamivudine
- Emtricitabine
- Efavirenz
- Zidovudine
- Tenofovir disoproxil fumarate (Tenofovir DF or TDF)
- Lopinavir/ritonavir
- Raltegravir
- Dolutegravir
- Darunavir
- Ritonavir

### Creating Analytic Datasets

This project aims to analyze Truvada utilization patterns for pre- and post-exposure prophylaxis at the patient- and prescribing practitioner-levels. This will require 4 analytic datasets:

|  | PrEP | PEP |
| --- | --- | --- |
| Person-level | 1 | 3 |
| Practitioner-level | 2 | 4 |

#### PrEP at patient-level data

I’ll use an algorithm for identifying persons prescribed tenofovir disoproxil fumarate and emtricitabine (TDF-FTC) for preexposure prophylaxis (PrEP) based on Figure 1 in the article by Wu et al (2016).


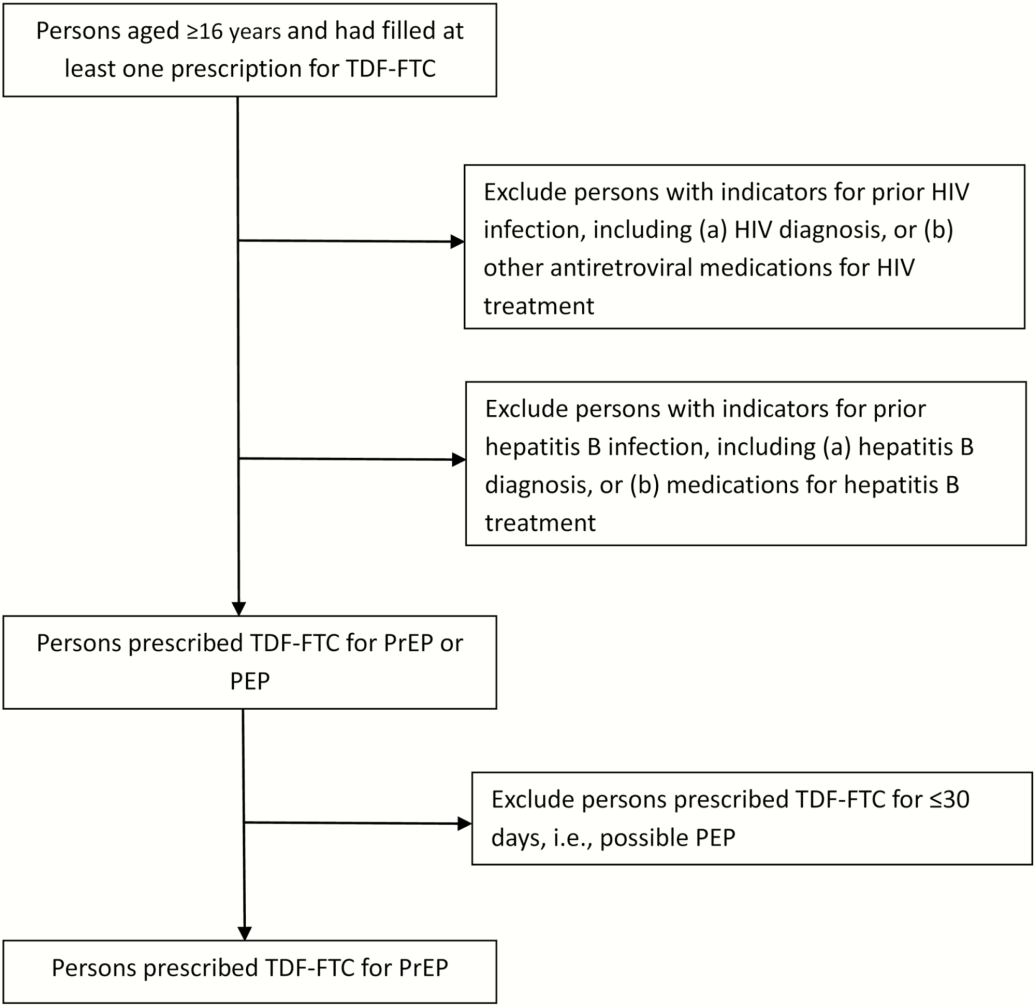


Hsiu Wu, Maria C. B. Mendoza, Ya-lin A. Huang, Tameka Hayes, Dawn K. Smith, Karen W. Hoover; Uptake of HIV Preexposure Prophylaxis Among Commercially Insured Persons—United States, 2010–2014. Clin Infect Dis 2017; 64 (2): 144-149. doi: 10.1093/cid/ciw701

Steps to creating the analytic dataset in SAS

1. Start with the [ Prescription claims file ]

| Patient ID | ClaimID | DrugID | PractitionerID | PlanID | … |
| --- | --- | --- | --- | --- | --- |
| 1 | 1 |  |  |  |  |
|  | 2 |  |  |  |  |
|  | 3 |  |  |  |  |
| 2 | 1 |  |  |  |  |
|  | 2 |  |  |  |  |
| … | … |  |  |  |  |
| n | m |  |  |  |  |

1. Merge with [ Patient file ] by PatientID to add variable: Age

| Patient ID | ClaimID | DrugID | PractitionerID | PlanID | … | Age |
| --- | --- | --- | --- | --- | --- | --- |
| 1 | 1 |  |  |  |  | 30 |
|  | 2 |  |  |  |  | 30 |
|  | 3 |  |  |  |  | 30 |
| 2 | 1 |  |  |  |  | 35 |
|  | 2 |  |  |  |  | 35 |
| … | … |  |  |  |  | … |
| n | m |  |  |  |  | … |

- 1. Create flag for age >= 16 yo

1. Merge with [ Drug file ] by DrugD to add variable:Drug_Name

| Patient ID | ClaimID | DrugID | PractitionerID | PlanID | … | Age | Drug Name |
| --- | --- | --- | --- | --- | --- | --- | --- |
| 1 | 1 |  |  |  |  | 30 |  |
|  | 2 |  |  |  |  | 30 |  |
|  | 3 |  |  |  |  | 30 |  |
| 2 | 1 |  |  |  |  | 35 |  |
|  | 2 |  |  |  |  | 35 |  |
| … | … |  |  |  |  | … |  |
| n | m |  |  |  |  | … |  |

- 1. Create flag for prescription claim for the drug Truvada

1. Merge with [Diagnosis claims file] by ClaimID and PatientID for the variable: diagnosis code

| Patient ID | ClaimID | DrugID | PractitionerID | PlanID | … | Age | Drug Name | Diagnosis Code | Flag: HIV infection | Flag: Hep B infection | Flag: Grand Total of Truvada supply days <= 30 |
| --- | --- | --- | --- | --- | --- | --- | --- | --- | --- | --- | --- |
| 1 | 1 |  |  |  |  | 30 |  | 1 |  |  |  |
|  | 2 |  |  |  |  | 30 |  | 1 |  |  |  |
|  |  |  |  |  |  |  |  | 2 |  |  |  |
|  | 3 |  |  |  |  | 30 |  | 1 |  |  |  |
|  |  |  |  |  |  |  |  | 2 |  |  |  |
|  |  |  |  |  |  |  |  | 3 |  |  |  |
| 2 | 1 |  |  |  |  | 35 |  | 1 |  |  |  |
|  | 2 |  |  |  |  | 35 |  | 1 |  |  |  |
| … | … |  |  |  |  | … |  | … |  |  |  |
| n | m |  |  |  |  | 65 |  | … |  |  |  |

- 1. Create flag for HIV diagnosis
  2. Create flag for HIV antiretroviral treatment
  3. Create flag for Hepatitis B diagnosis
  4. Create flag for hepatitis B treatment
  5. Generate new variable : total supply = days supply * quantity
     1. Generate Grand total supply for each patientID
     2. Create flag for grant total <= 30 days of supply

1. Collapse data at the Patient ID level
   1. Sum flags that were created 🡺 identify patients using Truvada for PrEP
   2. Sum number of claims for Truvada
   3. Keep ( all ) Practitioner IDs associated with patients
   4. Keep ( all ) plan IDs associated with patients

| Patient ID | PractitionerID | PlanID | Sum of Indicators for each patient | Flag: Patient is using Truvada for PrEP |
| --- | --- | --- | --- | --- |
| 1 | 1 | 1 |  |  |
| 2 |  |  |  |  |
| 3 | 2 |  |  |  |
| 4 | 3 | 2 |  |  |
| … |  |  |  |  |
| n | p |  |  |  |

1. Merge with [Patient File] by Patient ID for variables: patient demographics
2. Merge with [Practitioner File] by Practitioner ID for variables: practitioner info
3. Merge with [Plan File] by Plan ID for variables: plan info

| Patient ID | PractitionerID | PlanID | Flag: Patient is using Truvada for PrEP | Patient Demographics | Practitioner Info | Plan Info |
| --- | --- | --- | --- | --- | --- | --- |
| 1 | 1 | 1 |  |  |  |  |
| 2 |  | 2 |  |  |  |  |
| 3 | 2 |  |  |  |  |  |
| 4 | 3 |  |  |  |  |  |
| 5 | 4 | 3 |  |  |  |  |
| 6 |  | 4 |  |  |  |  |
| 7 |  | 5 |  |  |  |  |
| 8 |  |  |  |  |  |  |
| 9 | 5 | 6 |  |  |  |  |
| … | … | … |  |  |  |  |
| n | p | q |  |  |  |  |

#### PrEP at practitioner-level data

1. Collapse data at the Practitioner-level for patients using PrEP
   1. Calculate number of patients for each practitioner
   2. Calculate percent of patients with patient demographics for each practitioner
   3. Calculate percent of patients with plan info for each practitioner

| PractitionerID | Percent of Patient for each Practitioner that belongs to various Demographic categories | Percent of Patient for each Practitioner that belongs to various Plan categories |
| --- | --- | --- |
| 1 |  |  |
| 2 |  |  |
| 3 |  |  |
| 4 |  |  |
| … |  |  |
| p |  |  |

1. Merge with [Practitioner File] by Practitioner ID for variables; practitioner info

| PractitionerID | Percent of Patient for each Practitioner that belongs to various Demographic categories | Percent of Patient for each Practitioner that belongs to various Plan categories | Practitioner Info |
| --- | --- | --- | --- |
| 1 |  |  |  |
| 2 |  |  |  |
| 3 |  |  |  |
| 4 |  |  |  |
| … |  |  |  |
| p |  |  |  |

## Analysis

### Descriptive Statistics

Table 1: Demographic characteristics of persons prescribed **PrEP** and **PEP** from Sep 2015-Aug 2016

|  | PrEP | | PEP | |
| --- | --- | --- | --- | --- |
|  | Number of patients | Percentage of patients | Number of patients | Percentage of patients |
| Total |  |  |  |  |
| Age categories |  |  |  |  |
| … |  |  |  |  |
| Sex |  |  |  |  |
| .. |  |  |  |  |
| Ethnicity |  |  |  |  |
| … |  |  |  |  |
| Income |  |  |  |  |
| … |  |  |  |  |
| Education |  |  |  |  |
| … |  |  |  |  |
| Census Region |  |  |  |  |
| … |  |  |  |  |
| Info on Plans used by PrEP patients |  |  |  |  |
| … |  |  |  |  |

Table 2: Characteristics of practitioners who prescribed **PrEP** and **PEP** from Sep 2015-Aug 2016

|  | PrEP | | PEP | |
| --- | --- | --- | --- | --- |
|  | Number of practitioners | Percentage of practitioners | Number of practitioners | Percentage of practitioners |
| Total |  |  |  |  |
| State |  |  |  |  |
| … Top 10 states |  |  |  |  |
| Zip code |  |  |  |  |
| … Top 10 states |  |  |  |  |
| Specialty |  |  |  |  |
| … Top 10 specialties |  |  |  |  |

Figure 1: Histogram of prescribing physicians for **PrEP** across zip codes

Figure 2: Histogram of prescribing physicians for **PEP** across zip codes
