# Supplementary material for: Pre-exposure prophylaxis for preventing acquisition of HIV: A cross-sectional study of patients, prescribers, uptake, and spending in the United States, 2015–2016
Source: PLoS Med. 2020 Apr 10;17(4):e1003072. doi: 10.1371/journal.pmed.1003072 (PMC7147726; doi:10.1371/journal.pmed.1003072)
Supplement: S1 Appendix — This files contains the following: a) Table A, IDV prescription patient distribution by census region; b) Table B, demographic characteristics of individuals prescribed PrEP with commercial health insurance from September 2015–August 2016 from IDV data and individuals prescribed PrEP with commercial health insurance in 2014 from MarketScan Data; c) Table C, summary of chi-squared test results comparing the age, race/ethnicity, and sex composition between PrEP patients and HIV diagnosis; d) Table D, number of PrEP patients, number of diagnoses of HIV infection in 2015, and ratio of relative uptake of PrEP by MSA, ranked by ratio from highest to lowest for 107 MSAs; e) Table E, number of practitioners prescribing PrEP by specialty, September 2015–August 2016; f) Table F, top 20 diagnoses of PrEP patients; g) Table G, top 20 MSAs by number of PrEP-prescribing practitioners, September 2015–August 2016; h) Table H, average patient and third-party payor payments for PrEP by payment method category, September 2015–August 2016. IDV, Integrated Dataverse; MSA, metropolitan statistical area; PrEP, pre-exposure prophylaxis; STROBE, Strengthening the Reporting of Observational Studies in Epidemiology. (DOCX) [file pmed.1003072.s003.docx]

# Pre-exposure prophylaxis (PrEP) for preventing acquisition of HIV: A cross-sectional study of patients, prescribers, uptake, and spending in the United States, 2015-2016

### Table A: IDV Prescription Patient Distribution by Census Region

| **Census Region** | **IDV patients with at least one prescription** | | **U.S. Census Bureau** |
| --- | --- | --- | --- |
|  | Count | Percent |  |
| Northeast | 42,072,546 | 19.10% | 17.50% |
| Midwest | 48,461,360 | 22.00% | 21.13% |
| South | 83,276,360 | 37.81% | 36.81% |
| West | 43,605,610 | 19.80% | 23.10% |
| Other* | 2,817,909 | 1.28% | 1.46% |
| * APO/FPU, AS, GU, PR | |  |  |

### Table B: Demographic characteristics of individuals prescribed PrEP with Commercial Health Insurance from Sep 2015-Aug 2016 from IDV Data and individuals prescribed PrEP with Commercial Health Insurance in 2014 from MarketScan Data

| **Categories** | | **Number of PrEP Patients from IDV Data, Sep 2015 to Aug 2016** | **Percent** | **Number of PrEP Patients from MarketScan, 2014** | **Percent** |
| --- | --- | --- | --- | --- | --- |
| Total |  | 60,957 | 100.0 | 9375 | 100.0 |
| Age^ |  |  |  |  |  |
|  | 16-34 | 28,485 | 46.7 | 4,022 | 42.9 |
|  | 35-44 | 15,589 | 25.6 | 2,908 | 31.0 |
|  | 45-54 | 12,184 | 20.0 | 1,908 | 20.4 |
|  | 55-64 | 4,242 | 7.0 | 536 | 5.7 |
|  | >= 65 | 457 | 0.8 | Not Applicable |  |
| Ethnicity |  |  |  |  |  |
|  | Black/African American | 4,408 | 7.2 | Not Available |  |
|  | Hispanic | 5,161 | 8.5 | Not Available |  |
|  | White/Caucasian | 29,145 | 47.8 | Not Available |  |
|  | Other | 1,865 | 3.1 | Not Available |  |
|  | Unknown | 20,378 | 33.4 | Not Available |  |
| Census Region | |  |  |  |  |
|  | Midwest | 10,165 | 16.7 | 1,746 | 16.8 |
|  | Northeast | 15,683 | 25.7 | 1,247 | 13.3 |
|  | South | 18,882 | 31.0 | 2,336 | 24.9 |
|  | West | 15,845 | 26.0 | 4,043 | 43.1 |
|  | Other/Unknown | 382 | 0.6 | Not Applicable |  |
| Sex |  |  |  |  |  |
|  | Male | 58,486 | 96.0 | 9,137 | 97.5 |
|  | Female | 2,471 | 4.1 | 238 | 2.5 |
| Education | |  |  |  |  |
|  | HS Graduate Or Less | 10,318 | 16.9 | Not Available |  |
|  | Some College | 13,868 | 22.8 | Not Available |  |
|  | Associate Degree/Bachelor Degree Or More | 17,240 | 28.3 | Not Available |  |
|  | Unknown | 19,531 | 32.0 | Not Available |  |
| Income |  |  |  |  |  |
|  | Under 30K | 3,073 | 5.0 | Not Available |  |
|  | 30-49K | 6,356 | 10.4 | Not Available |  |
|  | 50-74K | 9,362 | 15.4 | Not Available |  |
|  | 75-99K | 7,440 | 12.2 | Not Available |  |
|  | 100K+ | 13,602 | 22.3 | Not Available |  |
|  | Unknown | 21,124 | 34.7 | Not Available |  |

PrEP=preexposure prophylaxis; HS=high school

*Note:* Another study analyzed PrEP utilization using the 2010-2014 MarketScan database,^[[1]](#footnote-1)^ which includes employer-sponsored insurance claims. We compared the subset of PrEP patients in the IDV database that used commercial health insurance to pay for PrEP with the results of the prior analysis as a way to check if this analysis is consistent with previous work. The comparison found PrEP patients in both databases were similar in sex and age, but had different distributions across Census regions. Given that both databases are based on convenience sampling, the geographic differences may simply reflect differences in the geographic locations of data contributors for each database, but we are unable to determine whether this is the case. Still, the similarities in the sex and age distributions of the PrEP patient populations in each of the databases gives added confidence that the differences observed between PrEP patients in the IDV database and those newly diagnosed with HIV are substantive.

^ Age categories for PrEP patients are 16-35, 36-45, 46-55, 55-65, Over 65

Table C. Summary of Chi-Square Test Results comparing the Age, Race/Ethnicity, and Sex composition between PrEP Patients and HIV diagnosis

| **MSA** | **Age** | **Race** | **Sex** |  | **Number of variables with p-values >= 0.05** |
| --- | --- | --- | --- | --- | --- |
| Akron, OH | 0.2819 | 0.0005 | 0.0022 |  | 1 |
| Albany-Schenectady-Troy, NY | 0.1096 | <.0001 | 0.1514 |  | 2 |
| Albuquerque, NM | 0.0127 | 0.0009 | 0.7441 |  | 1 |
| Allentown-Bethlehem-Easton, PA-NJ | 0.3689 | 0.0080 | 0.0039 |  | 1 |
| Atlanta-Sandy Springs-Roswell, GA | <.0001 | <.0001 | <.0001 |  | 0 |
| Augusta-Richmond County, GA-SC | 0.0416 | 0.0030 | 0.1163 |  | 1 |
| Austin-Round Rock, TX | <.0001 | <.0001 | <.0001 |  | 0 |
| Bakersfield, CA | 0.0887 | 0.0002 | 0.0793 |  | 2 |
| Baltimore-Columbia-Towson, MD | 0.4450 | <.0001 | <.0001 |  | 1 |
| Baton Rouge, LA | 0.0052 | <.0001 | 0.0097 |  | 0 |
| Birmingham-Hoover, AL | 0.5231 | <.0001 | 0.0016 |  | 1 |
| Boise City, ID | 0.4205 | 0.0001 | 0.0421 |  | 1 |
| Boston-Cambridge-Newton, MA-NH | 0.0103 | <.0001 | <.0001 |  | 0 |
| Bridgeport-Stamford-Norwalk, CT | 0.5035 | 0.0001 | <.0001 |  | 1 |
| Buffalo-Cheektowaga-Niagara Falls, NY | 0.1658 | <.0001 | 0.1071 |  | 2 |
| Cape Coral-Fort Myers, FL | 0.0050 | 0.0013 | 0.6571 |  | 1 |
| Charleston-North Charleston, SC | 0.0147 | <.0001 | 0.4141 |  | 1 |
| Charlotte-Concord-Gastonia, NC-SC | <.0001 | <.0001 | 0.0001 |  | 0 |
| Chattanooga, TN-GA | 0.2101 | <.0001 | 0.0538 |  | 2 |
| Chicago-Naperville-Elgin, IL-IN-WI | <.0001 | <.0001 | <.0001 |  | 0 |
| Cincinnati, OH-KY-IN | 0.0079 | <.0001 | 0.0005 |  | 0 |
| Cleveland-Elyria, OH | 0.0002 | <.0001 | 0.2096 |  | 1 |
| Colorado Springs, CO | 0.9047 | 0.0657 | 0.0765 |  | 3 |
| Columbia, SC | <.0001 | 0.0008 | 0.2784 |  | 1 |
| Columbus, OH | 0.0685 | <.0001 | <.0001 |  | 1 |
| Dallas-Fort Worth-Arling, TX | <.0001 | <.0001 | <.0001 |  | 0 |
| Dayton, OH | 0.5476 | <.0001 | 0.1330 |  | 2 |
| Deltona-Daytona Beach-Ormond Beach, FL | 0.1340 | 0.0033 | 0.3973 |  | 2 |
| Denver-Aurora-Lakewood, CO | 0.0464 | <.0001 | <.0001 |  | 0 |
| Des Moines-West Des Moines, IA | 0.9290 | <.0001 | 0.0024 |  | 1 |
| Detroit-Warren-Dearborn, MI | 0.0013 | <.0001 | 0.3322 |  | 1 |
| Durham-Chapel Hill, NC | 0.0250 | <.0001 | 0.3682 |  | 1 |
| El Paso, TX | 0.0270 | 0.0778 | 0.2660 |  | 2 |
| Fayetteville-Springdale-Rogers, AR-MO | 0.3990 | 0.5400 | 0.2874 |  | 3 |
| Fresno, CA | 0.2663 | <.0001 | 0.0049 |  | 1 |
| Grand Rapids-Wyoming, MI | 0.9911 | 0.0021 | 0.0624 |  | 2 |
| Greensboro-High Point, NC | 0.0002 | <.0001 | 0.2209 |  | 1 |
| Greenville-Anderson-Mauldin, SC | 0.1766 | 0.0003 | 0.1648 |  | 2 |
| Harrisburg-Carlisle, PA | 0.5503 | 0.0036 | 0.5710 |  | 2 |
| Hartford-West Hartford-East Hartford, CT | 0.0274 | <.0001 | <.0001 |  | 0 |
| Houston-The Woodlands-Sugar Land, TX | <.0001 | <.0001 | <.0001 |  | 0 |
| Indianapolis-Carmel-Anderson, IN | 0.0058 | <.0001 | 0.0014 |  | 0 |
| Jackson, MS | 0.0057 | <.0001 | 0.0004 |  | 0 |
| Jacksonville, FL | 0.0096 | <.0001 | 0.0013 |  | 0 |
| Kansas City, MO-KS | 0.0003 | <.0001 | 0.0314 |  | 0 |
| Knoxville, TN | 0.8113 | 0.1452 | <.0001 |  | 2 |
| Lakeland-Winter Haven, FL | 0.1719 | <.0001 | 0.5276 |  | 2 |
| Lancaster, PA | 0.9722 | 0.0427 | 0.4262 |  | 2 |
| Las Vegas-Henderson-Paradise, NV | 0.0534 | <.0001 | 0.0004 |  | 1 |
| Lexington-Fayette, KY | 0.1147 | 0.0020 | 0.1324 |  | 2 |
| Little Rock-North Little Rock-Conway, AR | 0.0197 | <.0001 | 0.0046 |  | 0 |
| Los Angeles-Long Beach-Anaheim, CA | <.0001 | <.0001 | <.0001 |  | 0 |
| Louisville/Jefferson County, KY-IN | 0.0590 | 0.0034 | <.0001 |  | 1 |
| Madison, WI | 0.3873 | <.0001 | 0.1712 |  | 2 |
| McAllen-Edinburg-Mission, TX | 0.5752 | <.0001 | 0.1298 |  | 2 |
| Memphis, TN-MS-AR | <.0001 | <.0001 | 0.0009 |  | 0 |
| Miami-Fort Lauderdale-WestPalm Beach, FL | <.0001 | <.0001 | <.0001 |  | 0 |
| Milwaukee-Waukesha-West Allis, WI | 0.0073 | <.0001 | 0.1436 |  | 1 |
| Minneapolis-St. Paul-Bloomington, MN-WI | 0.3764 | <.0001 | <.0001 |  | 1 |
| Nashville-Davidson--Murfreesboro--Frankl | <.0001 | <.0001 | 0.0148 |  | 0 |
| New Haven-Milford, CT | 0.6564 | <.0001 | 0.0006 |  | 1 |
| New Orleans-Metairie, LA | 0.0153 | <.0001 | <.0001 |  | 0 |
| New York-Jersey City, NY-NJ-PA | <.0001 | <.0001 | <.0001 |  | 0 |
| North Port-Sarasota-Bradenton, FL | 0.0732 | 0.0003 | 0.0453 |  | 1 |
| Oklahoma City, OK | 0.0255 | 0.0002 | 0.2810 |  | 1 |
| Omaha-Council Bluffs, NE-IA | 0.2393 | 0.0791 | 0.1115 |  | 3 |
| Orlando-Kissimmee-Sanford, FL | <.0001 | <.0001 | <.0001 |  | 0 |
| Oxnard-Thousand Oaks-Ventura, CA | 0.0051 | 0.3547 | 0.5377 |  | 2 |
| Palm Bay-Melbourne-Titusville, FL | 0.2544 | 0.0088 | 0.2084 |  | 2 |
| Philadelphia-Camden-Wilmingtaon, PA-NJ-DE-MD | 0.0027 | <.0001 | <.0001 |  | 0 |
| Phoenix-Mesa-Scottsdale, AZ | <.0001 | <.0001 | <.0001 |  | 0 |
| Pittsburgh, PA | 0.4264 | <.0001 | <.0001 |  | 1 |
| Portland-South Portland, ME | 0.7457 | <.0001 | <.0001 |  | 1 |
| Portland-Vancouver-Hillsboro, OR-WA | 0.6328 | 0.0002 | 0.0086 |  | 1 |
| Providence-Warwick, RI-MA | 0.7417 | <.0001 | 0.0007 |  | 1 |
| Raleigh, NC | <.0001 | <.0001 | <.0001 |  | 0 |
| Richmond, VA | <.0001 | <.0001 | 0.9611 |  | 1 |
| Riverside-San Bernardino-Ontario, CA | <.0001 | <.0001 | 0.0004 |  | 0 |
| Rochester, NY | 0.0696 | <.0001 | 0.0009 |  | 1 |
| Sacramento--Roseville--Arden-Arcade, CA | 0.0034 | <.0001 | 0.1657 |  | 1 |
| Salt Lake City, UT | 0.6922 | <.0001 | 0.0019 |  | 1 |
| San Antonio-New Braunfels, TX | <.0001 | <.0001 | 0.9231 |  | 1 |
| San Diego-Carlsbad, CA | <.0001 | <.0001 | <.0001 |  | 0 |
| San Francisco-Oakland-Hayward, CA | 0.1181 | <.0001 | <.0001 |  | 1 |
| San Jose-Sunnyvale-Santa Clara, CA | 0.0319 | <.0001 | 0.0004 |  | 0 |
| San Juan-Carolina-Caguas, PR | <.0001 |  | 0.0001 |  | 0 |
| Santa Rosa, CA | 0.0541 | 0.8528 | 0.2813 |  | 3 |
| Scranton--Wilkes-Barre--Hazleton, PA | 0.1546 | 0.0030 | 0.3330 |  | 2 |
| Seattle-Tacoma-Bellevue, WA | 0.0164 | <.0001 | <.0001 |  | 0 |
| Spokane-Spokane Valley, WA | 0.2392 | 0.3230 | 0.0331 |  | 2 |
| Springfield, MA | 0.7891 | 0.0392 | 0.4523 |  | 2 |
| St. Louis, MO-IL | <.0001 | <.0001 | <.0001 |  | 0 |
| Stockton-Lodi, CA | 0.1489 | 0.1311 | 0.4010 |  | 3 |
| Syracuse, NY | 0.4806 | 0.0014 | 0.6460 |  | 2 |
| Tampa-St. Petersburg-Clearwater, FL | <.0001 | <.0001 | <.0001 |  | 0 |
| Toledo, OH | 0.9339 | 0.0155 | 0.1472 |  | 2 |
| Tucson, AZ | 0.3149 | <.0001 | 0.0001 |  | 1 |
| Tulsa, OK | <.0001 | <.0001 | 0.0814 |  | 1 |
| Virginia Beach-Norfolk-Newport News, VA-NC | 0.0228 | 0.0001 | 0.3949 |  | 1 |
| Washington-Arlington-Alexandria, DC-VA-MD-WV | 0.0006 | <.0001 | <.0001 |  | 0 |
| Wichita, KS | 0.6744 | 0.0023 | 0.0083 |  | 1 |
| Winston-Salem, NC | <.0001 | <.0001 | 0.5595 |  | 1 |
| Worcester, MA-CT | 0.2001 | 0.0006 | 0.0790 |  | 2 |
| Youngstown-Warren-Boardman, OH-PA | 0.7297 | 0.4200 | 0.4474 |  | 3 |

PrEP=preexposure prophylaxis; MSA=metropolitan statistical area

Source: Authors’ analysis of the IDV data from September 2015 to August 2016, and Centers for Disease Control and Prevention’s (CDC) HIV Surveillance Report- Supplemental Report v22-1

Note: MSAs included have populations of 500,000 or greater, and greater than 20 newly diagnosed HIV infections. Age categories for PrEP patients are 16-35, 36-45, 46-55, 55+; whereas the age categories for Newly diagnosed HIV infections are 13-34, 35-44, 45-54, 55+. Race categories American Indian/Alaskan Natives and Asian were combined to create category ‘Other’ for Newly diagnosed HIV infections. PrEP patients whose race/ethnicity were ‘Unknown’ were not included in the chi-square test analysis. Sex categories are male and female.

### Table D. Number of PrEP Patients, Number of Diagnoses of HIV Infection in 2015, and Ratio of Relative Uptake of PrEP by MSA, Ranked by Ratio from Highest to Lowest for 107 MSAs

| **MSA** | **Number of PrEP Patients** | **Number of Diagnoses of HIV Infection, 2015** | **Ratio of Number of PrEP Patients to Number of Diagnoses of HIV Infection** | **Ranking of Ratio, from Highest to Lowest Approximate Uptake** |
| --- | --- | --- | --- | --- |
| Madison, WI | 204 | 21 | 9.71 | 1 |
| Seattle-Tacoma-Bellevue, WA | 2,877 | 334 | 8.61 | 2 |
| San Francisco-Oakland-Hayward, CA | 5,625 | 722 | 7.79 | 3 |
| Boston-Cambridge-Newton, MA-NH | 2,876 | 456 | 6.31 | 4 |
| Portland-Vancouver-Hillsboro, OR-WA | 897 | 165 | 5.44 | 5 |
| Des Moines-West Des Moines, IA | 169 | 32 | 5.28 | 6 |
| Salt Lake City, UT | 384 | 75 | 5.12 | 7 |
| Minneapolis-St. Paul-Bloomington, MN-WI | 1,212 | 265 | 4.57 | 8 |
| Albany-Schenectady-Troy, NY | 223 | 50 | 4.46 | 9 |
| Rochester, NY | 375 | 87 | 4.31 | 10 |
| Chicago-Naperville-Elgin, IL-IN-WI | 5,347 | 1,380 | 3.87 | 11 |
| Providence-Warwick, RI-MA | 405 | 107 | 3.79 | 12 |
| Pittsburgh, PA | 622 | 165 | 3.77 | 13 |
| Boise City, ID | 72 | 20 | 3.60 | 14 |
| New York-Jersey City, NY-NJ-PA | 12,402 | 3,563 | 3.48 | 15 |
| Durham-Chapel Hill, NC | 279 | 81 | 3.44 | 16 |
| Columbus, OH | 826 | 240 | 3.44 | 17 |
| San Jose-Sunnyvale-Santa Clara, CA | 534 | 156 | 3.42 | 18 |
| Washington-Arlington-Alexandria, DC-VA-MD-WV | 3,941 | 1,233 | 3.20 | 19 |
| Denver-Aurora-Lakewood, CO | 789 | 272 | 2.90 | 20 |
| New Haven-Milford, CT | 229 | 83 | 2.76 | 21 |
| Santa Rosa, CA | 80 | 29 | 2.76 | 22 |
| Austin-Round Rock, TX | 901 | 330 | 2.73 | 23 |
| San Diego-Carlsbad, CA | 1,297 | 479 | 2.71 | 24 |
| St. Louis, MO-IL | 736 | 291 | 2.53 | 25 |
| Portland-South Portland, ME | 63 | 25 | 2.52 | 26 |
| Ogden-Clearfield, UT | 40 | 16 | 2.50 | 27 |
| Knoxville, TN | 133 | 55 | 2.42 | 28 |
| Syracuse, NY | 100 | 42 | 2.38 | 29 |
| Springfield, MA | 122 | 53 | 2.30 | 30 |
| Omaha-Council Bluffs, NE-IA | 126 | 56 | 2.25 | 31 |
| Spokane-Spokane Valley, WA | 56 | 25 | 2.24 | 32 |
| Worcester, MA-CT | 105 | 47 | 2.23 | 33 |
| Harrisburg-Carlisle, PA | 95 | 43 | 2.21 | 34 |
| Modesto, CA | 26 | 12 | 2.17 | 35 |
| Albuquerque, NM | 142 | 66 | 2.15 | 36 |
| Philadelphia-Camden-Wilmingtaon, PA-NJ-DE-MD | 1,839 | 862 | 2.13 | 37 |
| Bridgeport-Stamford-Norwalk, CT | 144 | 68 | 2.12 | 38 |
| Los Angeles-Long Beach-Anaheim, CA | 4,795 | 2,324 | 2.06 | 39 |
| Hartford-West Hartford-East Hartford, CT | 202 | 99 | 2.04 | 40 |
| Colorado Springs, CO | 55 | 28 | 1.96 | 41 |
| Nashville-Davidson--Murfreesboro--Frankl | 425 | 217 | 1.96 | 42 |
| Provo-Orem, UT | 23 | 12 | 1.92 | 43 |
| Phoenix-Mesa-Scottsdale, AZ | 1,038 | 544 | 1.91 | 44 |
| Indianapolis-Carmel-Anderson, IN | 443 | 237 | 1.87 | 45 |
| Milwaukee-Waukesha-West Allis, WI | 227 | 130 | 1.75 | 46 |
| Dayton, OH | 115 | 66 | 1.74 | 47 |
| Sacramento--Roseville--Arden-Arcade, CA | 317 | 182 | 1.74 | 48 |
| Grand Rapids-Wyoming, MI | 94 | 54 | 1.74 | 49 |
| Kansas City, MO-KS | 297 | 175 | 1.70 | 50 |
| Fayetteville-Springdale-Rogers, AR-MO | 37 | 22 | 1.68 | 51 |
| Allentown-Bethlehem-Easton, PA-NJ | 71 | 46 | 1.54 | 52 |
| Riverside-San Bernardino-Ontario, CA | 653 | 450 | 1.45 | 53 |
| Oxnard-Thousand Oaks-Ventura, CA | 74 | 51 | 1.45 | 54 |
| Buffalo-Cheektowaga-Niagara Falls, NY | 187 | 130 | 1.44 | 55 |
| Atlanta-Sandy Springs-Roswell, GA | 2,417 | 1,689 | 1.43 | 56 |
| Cleveland-Elyria, OH | 315 | 229 | 1.38 | 57 |
| Tucson, AZ | 134 | 98 | 1.37 | 58 |
| Dallas-Fort Worth-Arling, TX | 1,707 | 1,275 | 1.34 | 59 |
| Akron, OH | 54 | 42 | 1.29 | 60 |
| Orlando-Kissimmee-Sanford, FL | 729 | 590 | 1.24 | 61 |
| Raleigh, NC | 186 | 154 | 1.21 | 62 |
| Toledo, OH | 37 | 31 | 1.19 | 63 |
| Lexington-Fayette, KY | 64 | 54 | 1.19 | 64 |
| Charlotte-Concord-Gastonia, NC-SC | 459 | 397 | 1.16 | 65 |
| Houston-The Woodlands-Sugar Land, TX | 1,588 | 1,455 | 1.09 | 66 |
| Birmingham-Hoover, AL | 189 | 176 | 1.07 | 67 |
| Wichita, KS | 60 | 58 | 1.03 | 68 |
| Scranton--Wilkes-Barre--Hazleton, PA | 38 | 37 | 1.03 | 69 |
| Tulsa, OK | 95 | 93 | 1.02 | 70 |
| Oklahoma City, OK | 161 | 158 | 1.02 | 71 |
| Miami-Fort Lauderdale-WestPalm Beach, FL | 2,189 | 2,276 | 0.96 | 72 |
| Chattanooga, TN-GA | 49 | 51 | 0.96 | 73 |
| Winston-Salem, NC | 65 | 68 | 0.96 | 74 |
| Cincinnati, OH-KY-IN | 194 | 210 | 0.92 | 75 |
| Lancaster, PA | 31 | 34 | 0.91 | 76 |
| New Orleans-Metairie, LA | 362 | 398 | 0.91 | 77 |
| Detroit-Warren-Dearborn, MI | 406 | 481 | 0.84 | 78 |
| Tampa-St. Petersburg-Clearwater, FL | 460 | 567 | 0.81 | 79 |
| Las Vegas-Henderson-Paradise, NV | 344 | 434 | 0.79 | 80 |
| Baltimore-Columbia-Towson, MD | 448 | 569 | 0.79 | 81 |
| Little Rock-North Little Rock-Conway, AR | 96 | 124 | 0.77 | 82 |
| Charleston-North Charleston, SC | 83 | 114 | 0.73 | 83 |
| Cape Coral-Fort Myers, FL | 57 | 84 | 0.68 | 84 |
| North Port-Sarasota-Bradenton, FL | 43 | 66 | 0.65 | 85 |
| Jackson, MS | 116 | 180 | 0.64 | 86 |
| Louisville/Jefferson County, KY-IN | 192 | 318 | 0.60 | 87 |
| Fresno, CA | 61 | 102 | 0.60 | 88 |
| Richmond, VA | 135 | 227 | 0.59 | 89 |
| San Antonio-New Braunfels, TX | 200 | 386 | 0.52 | 90 |
| Greensboro-High Point, NC | 67 | 131 | 0.51 | 91 |
| Greenville-Anderson-Mauldin, SC | 39 | 78 | 0.50 | 92 |
| Columbia, SC | 81 | 164 | 0.49 | 93 |
| Youngstown-Warren-Boardman, OH-PA | 15 | 35 | 0.43 | 94 |
| Jacksonville, FL | 131 | 315 | 0.42 | 95 |
| El Paso, TX | 48 | 116 | 0.41 | 96 |
| Stockton-Lodi, CA | 28 | 68 | 0.41 | 97 |
| Memphis, TN-MS-AR | 127 | 312 | 0.41 | 98 |
| Lakeland-Winter Haven, FL | 43 | 106 | 0.41 | 99 |
| Bakersfield, CA | 49 | 121 | 0.40 | 100 |
| Augusta-Richmond County, GA-SC | 41 | 104 | 0.39 | 101 |
| Palm Bay-Melbourne-Titusville, FL | 21 | 57 | 0.37 | 102 |
| San Juan-Carolina-Caguas, PR | 144 | 399 | 0.36 | 103 |
| Deltona-Daytona Beach-Ormond Beach, FL | 22 | 78 | 0.28 | 104 |
| Baton Rouge, LA | 54 | 265 | 0.20 | 105 |
| Virginia Beach-Norfolk-Newport News, VA-NC | 53 | 293 | 0.18 | 106 |
| McAllen-Edinburg-Mission, TX | 9 | 82 | 0.11 | 107 |

PrEP=preexposure prophylaxis; MSA=metropolitan statistical area.

### Table E. Number of Practitioners Prescribing PrEP by Specialty, September 2015-August 2016

| **Practitioner’s Specialty** | **Total Number of PrEP Patients** | **Percent of All Practitioners Prescribing PrEP** |
| --- | --- | --- |
| INTERNAL MEDICINE | 27,578 | 27.8 |
| FAMILY MEDICINE | 25,706 | 25.9 |
| FAMILY PRACTICE | 13,941 | 14.1 |
| INFECTIOUS DISEASES | 13,466 | 13.6 |
| PHYSICIAN ASSISTANT | 3,659 | 3.7 |
| EMERGENCY MEDICINE | 1,402 | 1.4 |
| NURSE PRACTITIONER | 1,310 | 1.3 |
| GYNECOLOGY | 1,232 | 1.2 |
| UNKNOWN | 1,213 | 1.2 |
| INTERNAL MEDICINE/PEDIATRICS | 1,156 | 1.2 |
| PEDIATRICS | 687 | < 1 |
| CRITICAL CARE MEDICINE | 680 | < 1 |
| GENERAL PRACTICE | 675 | < 1 |
| INTERNAL MEDICINE, GERIATRICS | 539 | < 1 |
| GASTROENTEROLOGY | 520 | < 1 |
| GENERAL PREVENTIVE MEDICINE | 480 | < 1 |
| OBSTETRICS AND GYNECOLOGY | 436 | < 1 |
| GENERAL SURGERY | 310 | < 1 |
| HOSPITALIST | 284 | < 1 |
| PSYCHIATRY | 249 | < 1 |

### Table F. Top 20 Diagnoses of PrEP Patients

| **Obs** | **Diagnosis** | **Diagnosis Code** | **Count** | **Percent** |
| --- | --- | --- | --- | --- |
| 1 | ENCOUNTER FOR GENERAL ADULT MEDICAL EXAMINATION WITHOUT ABNORMAL FINDINGS | Z00.00 | 8,354 | 2.58 |
| 2 | ENCOUNTER FOR IMMUNIZATION | Z23 | 7,494 | 2.32 |
| 3 | ENCOUNTER FOR SCREENING FOR INFECTIONS WITH A PREDOMINANTLY SEXUAL MODE OF TRANSMISSION | Z11.3 | 7,420 | 2.30 |
| 4 | CONTACT WITH AND (SUSPECTED) EXPOSURE TO HUMAN IMMUNODEFICIENCY VIRUS [HIV] | Z20.6 | 6,340 | 1.96 |
| 5 | CONTACT WITH AND (SUSPECTED) EXPOSURE TO INFECTIONS WITH A PREDOMINANTLY SEXUAL MODE OF TRANSMISSION | Z20.2 | 5,190 | 1.62 |
| 6 | HIGH RISK HETEROSEXUAL BEHAVIOR | Z72.51 | 4,305 | 1.34 |
| 7 | OTHER LONG TERM (CURRENT) DRUG THERAPY | Z79.899 | 4,110 | 1.27 |
| 8 | ESSENTIAL (PRIMARY) HYPERTENSION | I10 | 4,094 | 1.28 |
| 9 | HIGH RISK HOMOSEXUAL BEHAVIOR | Z72.52 | 3,393 | 1.06 |
| 10 | ANXIETY DISORDER, UNSPECIFIED | F41.9 | 2,472 | 0.76 |
| 11 | CONTACT WITH AND (SUSPECTED) EXPOSURE TO OTHER VIRAL COMMUNICABLE DISEASES | Z20.828 | 2,454 | 0.76 |
| 12 | ACUTE PHARYNGITIS, UNSPECIFIED | J02.9 | 2,277 | 0.70 |
| 13 | HYPERLIPIDEMIA, UNSPECIFIED | E78.5 | 2,165 | 0.67 |
| 14 | OBSTRUCTIVE SLEEP APNEA (ADULT) (PEDIATRIC) | G47.33 | 1,903 | 0.59 |
| 15 | MAJOR DEPRESSIVE DISORDER, SINGLE EPISODE, UNSPECIFIED | F32.9 | 1,889 | 0.58 |
| 16 | LOW BACK PAIN | M54.5 | 1,872 | 0.58 |
| 17 | TESTICULAR HYPOFUNCTION | E29.1 | 1,801 | 0.56 |
| 18 | ENCOUNTER FOR SCREENING FOR HUMAN IMMUNODEFICIENCY VIRUS [HIV] | Z11.4 | 1,687 | 0.53 |
| 19 | GASTRO-ESOPHAGEAL REFLUX DISEASE WITHOUT ESOPHAGITIS | K21.9 | 1,657 | 0.52 |
| 20 | VITAMIN D DEFICIENCY, UNSPECIFIED | E55.9 | 1,610 | 0.50 |

PrEP=preexposure prophylaxis

### Table G. Top 20 MSAs by Number of PrEP Prescribing Practitioners, September 2015-August 2016

| **MSA** | **Number of Practitioners** | **Percent of All Practitioners Who Prescribed PrEP** |
| --- | --- | --- |
| New York-Newark-Jersey City, NY-NJ-PA | 2,680 | 11.19% |
| Los Angeles-Long Beach-Anaheim, CA | 1,284 | 5.4% |
| San Francisco-Oakland-Hayward, CA | 936 | 3.9% |
| Chicago-Naperville-Elgin, IL-IN_WI | 914 | 3.8% |
| Boston-Cambridge-Newton, MA-NH | 834 | 3.5% |
| Washington-Arlington-Alexandria, DC-VA-MD-WV | 759 | 3.2% |
| Philadelphia-Camden-Wilmington, PA-NJ-DE-MD | 670 | 2.8% |
| Seattle-Tacoma-Bellevue, WA | 660 | 2.8% |
| Miami-Fort Lauderdale-West Palm Beach, FL | 579 | 2.4% |
| Portland-Vancouver-Hillsboro, OR-WA | 456 | 1.9% |
| Atlanta-Sandy Springs-Roswell, GA | 429 | 1.8% |
| Dallas-Fort Worth-Arlington, TX | 417 | 1.7% |
| San Diego-Carlsbad, CA | 394 | 1.6% |
| Houston-The Woodlands-Sugar Land, TX | 385 | 1.6% |
| Phoenix-Mesa-Scottsdale, AZ | 363 | 1.5% |
| Denver-Aurora-Lakewood, CO | 291 | 1.2% |
| Austin-Round Rock, TX | 264 | 1.1% |
| Minneapolis-St. Paul-Bloomington, MN-WI | 261 | 1.1% |
| Columbus, OH | 198 | 0.8% |
| Orlando-Kissimmee-Sanford, FL | 198 | 0.8% |

PrEP=preexposure prophylaxis; MSA=metropolitan statistical area

Table H. Average Patient and Third-Party Payer Payments for PrEP – by Payment Method Category, September 2015 through August 2016

| **Payment Method** | **Number of PrEP Users-Months** | **Patient and Third-Party Payor (TPP)** | **Average Monthly Payment** | **Std Dev** | **Minimum** | **Maximum** | **Third-Party Payor Share of Monthly Payment** | **Projected Yearly Payment** |
| --- | --- | --- | --- | --- | --- | --- | --- | --- |
| Any Insurance | 264,929 | Patient | $66 | $248 | $0 | $12,672 |  | $791 |
|  |  | TPP | $1,668 | $1,082 | $0 | $21,117 | 96% | $20,017 |
|  |  |  |  |  |  |  |  |  |
| Commercial | 187,148 | Patient | $77 | $231 | $0 | $9,682 |  | $925 |
|  |  | TPP | $1,868 | $1,109 | $0 | $21,117 | 96% | $22,411 |
|  |  |  |  |  |  |  |  |  |
| Medicaid | 30,580 | Patient | $5 | $45 | $0 | $2,949 |  | $55 |
|  |  | TPP | $1,578 | $581 | $0 | $8,969 | 100% | $18,931 |
|  |  |  |  |  |  |  |  |  |
| Medicare | 13,954 | Patient | $60 | $172 | $0 | $1,860 |  | $723 |
|  |  | TPP | $1,551 | $638 | $0 | $8,871 | 96% | $18,607 |
|  |  |  |  |  |  |  |  |  |
| Tricare | 2,013 | Patient | $17 | $15 | $0 | $144 |  | $201 |
|  |  | TPP | $1,648 | $720 | $434 | $8,379 | 99% | $19,772 |
|  |  |  |  |  |  |  |  |  |
| Gilead | 27,737 | Patient | $18 | $158 | $0 | $4,771 |  | $218 |
|  |  | TPP | $561 | $781 | $0 | $6,456 | 97% | $6,738 |
|  |  |  |  |  |  |  |  |  |
| Cash Only | 118 | Patient | $2,433 | $1,916 | $0 | $10,594 |  | $29,197 |
|  |  | TPP | $0 | $0 | $0 | $0 | 0% | $0 |
|  |  |  |  |  |  |  |  |  |
| Other Assistance | 3,281 | Patient | $305 | $940 | $0 | $12,672 |  | $3,662 |
|  |  | TPP | $1,104 | $894 | $0 | $8,865 | 78% | $13,249 |
|  |  |  |  |  |  |  |  |  |

1. Wu, H., Mendoza, M. C., Huang, Y. L. A., Hayes, T., Smith, D. K., & Hoover, K. W. (2016). Uptake of HIV preexposure prophylaxis among commercially insured individuals—United States, 2010–2014. *Clinical Infectious Diseases*, *64*(2), 144-149., <https://academic.oup.com/cid/article-abstract/64/2/144/2698875/Uptake-of-HIV-Preexposure-Prophylaxis-Among> [↑](#footnote-ref-1)
